# The salt-responsive transcriptome of chickpea roots and nodules via deepSuperSAGE

**DOI:** 10.1186/1471-2229-11-31

**Published:** 2011-02-14

**Authors:** Carlos Molina, Mainassara Zaman-Allah, Faheema Khan, Nadia Fatnassi, Ralf Horres, Björn Rotter, Diana Steinhauer, Laurie Amenc, Jean-Jacques Drevon, Peter Winter, Günter Kahl

**Affiliations:** 1 Molecular BioSciences, Biocenter, Johann Wolfgang Goethe University, Max-von-Laue-Str. 9, D-60439 Frankfurt am Main, Germany; 2 GenXPro GmbH, Frankfurt Innovation Center FIZ Biotechnology, Altendörferallee 3, D-60438 Frankfurt am Main, Germany; 3 Soil Symbiosis and Environment, INRA, 1 place Viala, 34060 Montpellier-Cedex, France; 4 Molecular Ecology Laboratory, Department of Botany, Jamia Hamdard University, New Delhi, India; 5 Estación Experimental del Zaidín, CSIC, C/Profesor Albareda, 1, 18008-Granada, Spain; 6 Unité de Recherche en Légumineuses, INRA-URLEG, 17 Rue Sully, 21000 Dijon, France

## Abstract

**Background:**

The combination of high-throughput transcript profiling and next-generation sequencing technologies is a prerequisite for genome-wide comprehensive transcriptome analysis. Our recent innovation of deepSuperSAGE is based on an advanced SuperSAGE protocol and its combination with massively parallel pyrosequencing on Roche's 454 sequencing platform. As a demonstration of the power of this combination, we have chosen the salt stress transcriptomes of roots and nodules of the third most important legume crop chickpea (*Cicer arietinum *L.). While our report is more technology-oriented, it nevertheless addresses a major world-wide problem for crops generally: high salinity. Together with low temperatures and water stress, high salinity is responsible for crop losses of millions of tons of various legume (and other) crops. Continuously deteriorating environmental conditions will combine with salinity stress to further compromise crop yields. As a good example for such stress-exposed crop plants, we started to characterize salt stress responses of chickpeas on the transcriptome level.

**Results:**

We used deepSuperSAGE to detect early global transcriptome changes in salt-stressed chickpea. The salt stress responses of 86,919 transcripts representing 17,918 unique 26 bp deepSuperSAGE tags (UniTags) from roots of the salt-tolerant variety INRAT-93 two hours after treatment with 25 mM NaCl were characterized. Additionally, the expression of 57,281 transcripts representing 13,115 UniTags was monitored in nodules of the same plants. From a total of 144,200 analyzed 26 bp tags in roots and nodules together, 21,401 unique transcripts were identified. Of these, only 363 and 106 specific transcripts, respectively, were commonly up- or down-regulated (>3.0-fold) under salt stress in both organs, witnessing a differential organ-specific response to stress.

Profiting from recent pioneer works on massive cDNA sequencing in chickpea, more than 9,400 UniTags were able to be linked to UniProt entries. Additionally, gene ontology (GO) categories over-representation analysis enabled to filter out enriched biological processes among the differentially expressed UniTags. Subsequently, the gathered information was further cross-checked with stress-related pathways.

From several filtered pathways, here we focus exemplarily on transcripts associated with the generation and scavenging of reactive oxygen species (ROS), as well as on transcripts involved in Na^+ ^homeostasis. Although both processes are already very well characterized in other plants, the information generated in the present work is of high value. Information on expression profiles and sequence similarity for several hundreds of transcripts of potential interest is now available.

**Conclusions:**

This report demonstrates, that the combination of the high-throughput transcriptome profiling technology SuperSAGE with one of the next-generation sequencing platforms allows deep insights into the first molecular reactions of a plant exposed to salinity. Cross validation with recent reports enriched the information about the salt stress dynamics of more than 9,000 chickpea ESTs, and enlarged their pool of alternative transcripts isoforms.

As an example for the high resolution of the employed technology that we coin deepSuperSAGE, we demonstrate that ROS-scavenging and -generating pathways undergo strong global transcriptome changes in chickpea roots and nodules already 2 hours after onset of moderate salt stress (25 mM NaCl). Additionally, a set of more than 15 candidate transcripts are proposed to be potential components of the salt overly sensitive (SOS) pathway in chickpea.

Newly identified transcript isoforms are potential targets for breeding novel cultivars with high salinity tolerance. We demonstrate that these targets can be integrated into breeding schemes by micro-arrays and RT-PCR assays downstream of the generation of 26 bp tags by SuperSAGE.

## Background

High salinity, together with low temperatures and water stress, are responsible for the large margin existing between the potential yield in tons hectar^-1 ^and the real harvest yield in several crops worldwide [1]. In semi-arid agricultural areas of the world, soil salinization is tightly linked to the extensive use of artificial irrigation, which in combination with extended dry seasons, very quickly turns formerly productive areas practically into desserts [2]. In the future, this effect will even increase due to the high demand of water from other non-agriculture sectors (i.e. industry, overpopulated cities), whereas the possibilities to increase any crop's productivity through irrigation will necessarily decrease [3,4]. Despite the remarkable ability of plants to cope with a wide range of stresses, the race against the continuously deteriorating environmental conditions on our planet will be lost unless new plant breeding strategies for abiotic stress-tolerance are developed.

Chickpea, one of the most important staple food legume crops worldwide, is cultivated in regions considered to be "the eye of the hurricane" in view of the adverse conditions like poor-watered and saline soils (Mediterranean basin, Indian sub-continent)[5]. The increasing demand of production, and the adaptation of this crop to less appropriate, even poor soils, forces to study the high salinity response mechanisms of this important non-model plant.

Plants under salt stress have to battle against two severe impacts: i) the ionic disequilibrium, caused by the increased amount of sodium in the soil; and ii) the osmotic misbalance, in which the osmotic potential of the soil drastically decreases [6,7]. Additionally, the metabolic alterations and high demand of energy caused by the first two stresses are leading to a third and sometimes more lethal obstacle: the oxidative stress [8]. As a consequence, salinity tolerance is expected to depend on genes encoding proteins 1) limiting the rate of Na^2+ ^uptake from the soil and managing its transport throughout the plant, 2) adjusting the ionic and osmotic balance of cells in roots and shoots, 3) regulating leaf development and the onset of senescence, and (4) controlling the overproduction of reactive oxygen species (superoxide [O_2_^-^], hydrogen peroxide [H_2_O_2_], and hydroxyl radicals [OH^-^])[9].

In model plants, extensive knowledge of biochemical and molecular processes underlying salt-stress responses has been accumulated over the past decades. Among several other striking advances in *Arabidopsis thaliana*, the signal transduction components of the salt overly sensitive pathway (SOS), a cascade activated by ionic disequilibrium, have been extensively characterized [10-13]. Further on, the activation of a specific salt-responsive MAP-kinase signalling cascade (the MKK2-MPK4-MPK6 pathway) has been uncovered [14]. Several studies of calcium-dependent protein kinases (CDPKs), a kinase family linked to stress signalling, revealed the mechanisms of Ca^2+ ^as messenger molecule in plants under stress alarm [15-17]. The dynamics of the transcriptome associated with ROS equilibrium (ROS production and detoxification) in plants has also been under intense scrutiny. For example, in *Arabidopsis *ROS-driven expression profiles on microarrays demonstrated that at least 8,000, out of 26,000 evaluated transcripts, changed their expression level upon ROS induction [18].

In sharp contrast to the importance of chickpeas as staple food and industrial raw material, the salt-responses at the transcriptome and proteome levels had only been dealt with at very low throughput until some years ago, i.e. tens, or at the most, hundreds of genes had been considered [19,20]. In the last couple of years, massive sequencing approaches made it possible to gather information from thousands of complete ESTs, extending the available sequence information for previously under-studied organisms. For chickpea, a pioneer work has already started to uncover large portions of the transcriptome under abiotic stress, increasing the number of ESTs sequences deposited in the public domain up to more than 20,000 entries [21].

In the present work we profit from the high resolution power of SuperSAGE coupled to the Roche 454 Life/APG GS FLX Titanium NGS technology to characterize the complete transcriptome of salt-stressed chickpea plants, especially at the onset of the stress. Here we report on 86,919 transcripts representing 17,918 unique 26 bp tags from roots of the salt-tolerant variety INRAT-93, 2 hours after 25 mM NaCl-treatment. In parallel, the expression of 57,281 transcripts grouped in 13'115 UniTags was monitored in nodules of the same plants. Only a total of 363 and 106 transcripts, respectively, were commonly up- or down-regulated (>3.0-fold) under salt stress in both organs, suggesting a strong organ-specific differential response upon salt stress.

Using the information generated by recent massive cDNA sequencing in chickpea, more than 14,000 of the obtained 26 bp tags were validated by ESTs deposited in the public domain, adding valuable information in terms of i) their dynamics in the tested variety and under experimental conditions, ii) their differential expression in roots and nodules of the same plant towards salt stress, and iii) the existence of large sets of very similar alternative transcript isoforms detected in the form of SNPs-associated alternative tags (here denoted as SAATs) [22].

After EST-bridged UniTags annotation to Fabaceae and Arabidopsis mRNAs, more than 9,400 UniTags could be linked to UniProt entries. Further on, Gene ontology (GO) categories over-representation analysis enabled us to filter out enriched biological processes among the differentially expressed UniTags in chickpea roots and nodules under salt stress. Subsequently, the gathered information was cross-checked with stress-related pathways for finer selection of potential transcripts of interest.

The vast amount of information generated here forced us to focus on transcripts associated with the generation and scavenging of reactive oxygen species as well as on transcripts associated with the maintenance of Na^+ ^homeostasis, as example scenarios where intense transcriptome-remodelling is occurring after stress onset. Nevertheless the present work opens also several gates for the possible identification of new genes related to other pathways, and the incorporation of previously not stress-associated genes into the salt-stress context.

## Results

### Abundance of 26 bp tags

A total of 144,200 26 bp tags from roots (86,919) and nodules (57,281), respectively, of the salt-tolerant variety INRAT-93 were sequenced from untreated plants (control) and plants treated with 25 mM NaCl for 2 h. After grouping the sequenced tags, a total of 17,918 and 13,115 unique transcripts (UniTags) were extracted from roots and nodules, respectively (excluding singletons). The expression profiles of 21,401 UniTags from both organs were revealed.

In roots, less than 1% percent of the 26 bp tags were present in very high copy numbers (>500 copies × 100,000^-1^), whereas 9% and 90% of the transcripts were present between 10 to 100 and less than 10 copies × 100,000^-1^, respectively. Similarly, in nodules of the same INRAT-93 plants, less than 1% of the transcripts were present in very high copy numbers (> 500 copies × 100,000^-1^). However, the number of transcripts in the different abundancy classes (10 to 100, and less than 10 copies × 100,000^-1^) varied to some extent. Fifteen percent fell in between 10 and 100 copies × 100,000^-1^, contrasting the 10% found in roots. Transcripts detected in less than 10 copies × 100,000^-1 ^made up ~ 85% of the total 26 bp tags. UniTags from control and stress libraries were deposited in the Gene Expression Omnibus (GEO) public domain under the series GSE26638.

### EST-bridged annotation of UniTags: mutualism between two profiling techniques

During the past years, the arrival of massive sequencing approaches enabled the sequencing of very large transcriptome portions for very favourable costs in relation to output. As a consequence, several groups have already started sequencing hundred thousands of complete cDNAs for species from which almost no sequence information was available. To prove the potential of combining the high quantitative resolution of a tagging technique with the high sequence quality obtained by large mRNA sequencing procedures, SuperSAGE libraries were annotated by linking UniTags to the more than 20,000 chickpea ESTs deposited in the public domain (plus additional 20,000 primary source sequences)[21]. After UniTag-linking, each EST sequence was re-annotated to Fabaceae and Arabidopsis databases obtained from NCBI (http://blast.ncbi.nlm.nih.gov) and TIGR (http://compbio.dfci.harvard.edu/).

From 21,401 UniTags (21,090 non-low complexity sequences), 14,423 found high homology matches with 8,837 chickpea ESTs. Through re-annotation of the ESTs to public databases, a total of 9,667 UniTags were assigned to 4,336 UniProt characterized entries. A total of 7,639 UniTags were linked to Gene Ontology (GO) terms (http://www.geneontology.org).

**Figure 1:**
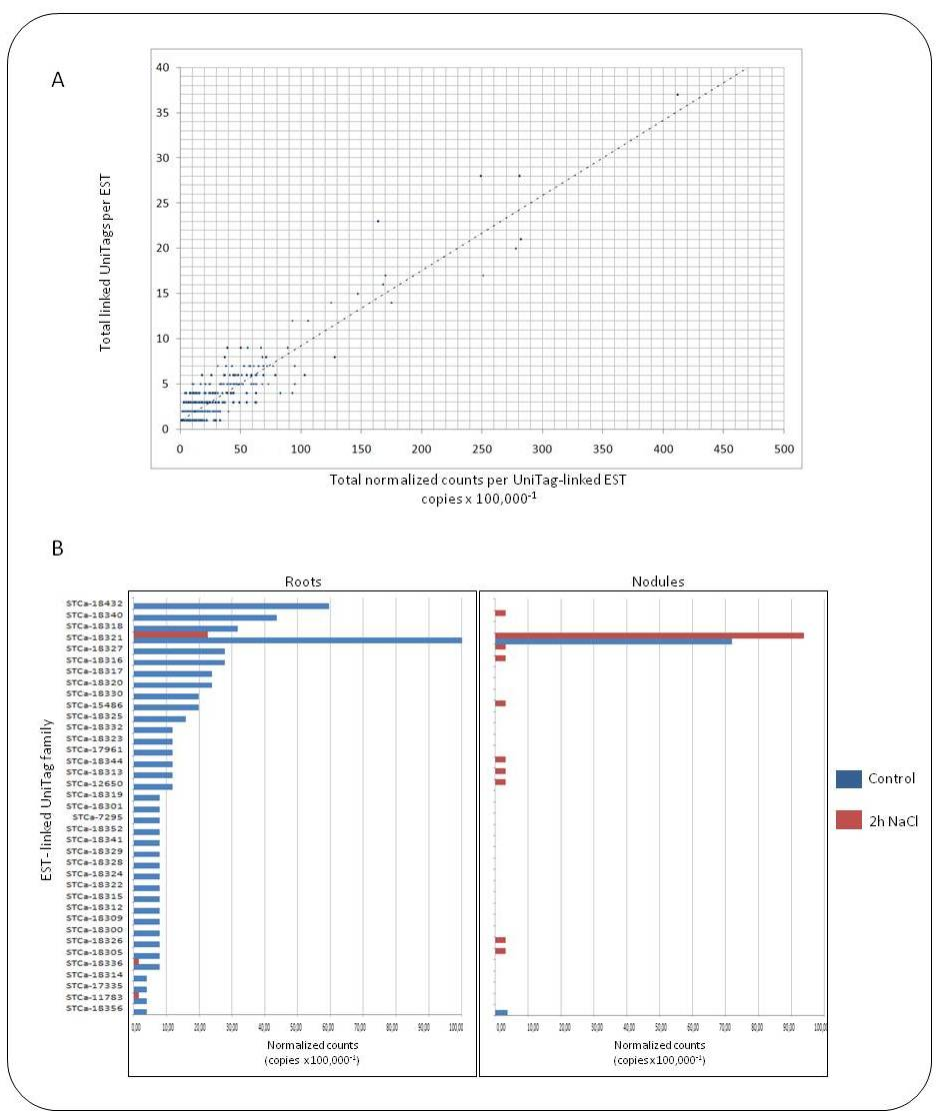
**Representation of chickpea ESTs in relation to homologous SuperSAGE UniTags**. Chickpea UniTags showed a broad range of copy numbers and homologies to ESTs already deposited in the public domain. Several single ESTs were targeted by more than one UniTag. As expected, the addition of copy numbers/EST was larger for ESTs targeted by several UniTags, than for ESTs targeted only by one 26 bp Tag. However, interestingly, the distribution of copy numbers is not equal across all the UniTags targeting the same EST. Very abundant UniTags are frequently accompanied by highly similar 26 bp tags found in lower copy numbers. A) Correlation between the number of chickpea UniTags and their accumulative copy numbers per target EST B) Distribution of copy numbers across a family of 37 UniTags hitting the same chickpea EST annotated to UniProt accession Q9LIN9_ARATH (unknown protein)

Concerning UniTag-to-EST representation, 6,283 out of 8,837 ESTs (71%) were represented by a single UniTag, whereas 1,636 (18%), 463 (5,2%), 181 (2,0%), 81 (0,9%) and 193 (2,18%) where represented by 2, 3, 4, 5 and >5 UniTags, respectively. Remarkably, the EST targeted by the largest number of UniTags was the Contig17642 (Q9LIN9_ARATH, 37 similar UniTags). For the total dataset, a positive correlation was found between the cumulative number of UniTag copies and the number of targeting UniTags for a given target EST (Figure 1). However, the distribution of copy numbers was not equal along all UniTags grouped to the same EST (families). Large families usually showed a single UniTag with high copy numbers accompanied by several similar tags found in much lower proportion (Figure 1).

### SNPs-associated alternative tags in SuperSAGE libraries

To assess the UniTags sequence similarity within chickpea SuperSAGE libraries without comparing to external ESTs, the datasets Ca-I93-NaCl-Ct and Ca-I93-NaCl-Str (control and salt-stressed roots, respectively) were self-BLASTed via stand alone BLAST [23]. Additionally, a SuperSAGE dataset from *Musa acuminata *(GPL2542) was retrieved from the gene expression omnibus (GEO, http://www.ncbi.nlm.nih.gov/geo) and self-BLASTed [24]. In this way, groups of UniTags sharing high sequence similarities were formed (excluding low complexity tags).

**Figure 2:**
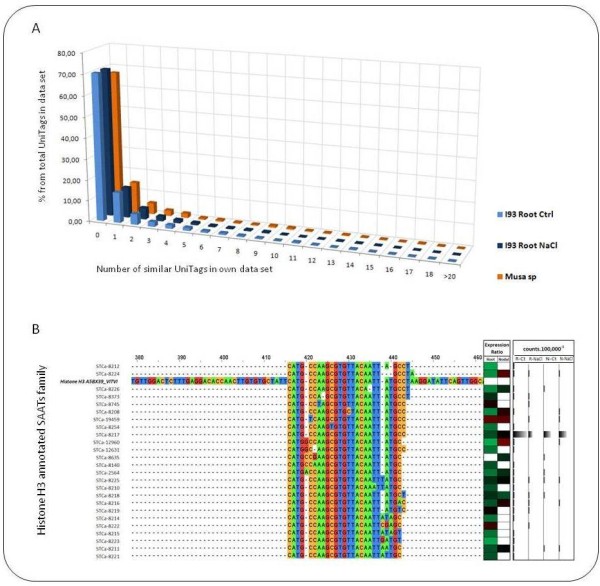
**Occurrence of highly similar UniTags within deepSuperSAGE libraries**. A) Proportion of similar hits found after BLASTing any given UniTag against its own SuperSAGE library. Three sources of UniTags were compared, comprising two chickpea SuperSAGE libraries and a *Musa acuminata *SuperSAGE library deposited in the public domain. Almost 70% of the UniTags do not find similar hits, whereas 30% can find more than one similar UniTag within the own library B) Example of a family of very similar UniTags annotated to a histone H3 UniProt entry. Several of the UniTags are differentiated by SNPs, and represent so called SNP-associated alternative tags (SAATs) families. Large copy number differences can be observed among very similar UniTags (graphically represented in the right panel)

Along the three evaluated SuperSAGE datasets, 70% of the UniTags did not find high homologies (>22 bp) to any other UniTag within the own library (Figure 2). In much lower proportions, 15, 4, and 2% of the UniTags, found one, two, and more than three similar hits, respectively, within the own libraries. Several of the similar UniTags belonging to the same family were differentiated by SNPs, a phenomenon already reported for humans, and known as SNPs-associated alternative tags (here denoted as, SAATs) [22]. An example of a large SAAT family of chickpea UniTags is depicted in Figure 2.

### Diversity of expression profiles along SAATs

As exemplified in Figure 2 through a family of 26 UniTags annotated to a histone H3 protein (A5BX39_VITVI), UniTags associated to the same SAAT family very often showed different expression profiles and a very different distribution of copy numbers. For the exemplified case, UniTag STCa-8217 showed the largest number of copies in all four libraries with a total of 2,026.78 copies × 100,000^-1^, whereas the remaining 25 UniTags only added up to 316.47 copies × 100,000^-1^. Remarkably, the majority of significant expression changes are seen in low copy number UniTags. The present result emphasizes the complementarities of both SuperSAGE and other massive sequencing approaches. In SuperSAGE libraries all sequencing efforts are directed to a discrete region of a given EST, thus gaining on resolution in a determined region, however, sacrificing the larger coverage that could be obtained through larger reads (e.g. RNAseq libraries).

### Stress- and organ-related differential gene expression in chickpea roots and nodules

In roots of the salt-tolerant chickpea variety INRAT-93, 35% of the 26 bp tags were at least 2.7-fold up- or down-regulated [R_(ln)_>1.0], respectively, after only 2 hours of exposure to 25 mM NaCl. From these, more than 2,000 tags (11%) were at least 8-fold down-regulated, a much higher proportion than the mere 1.93% (346 tags) showing more than 8-fold up-regulation in the same organ, and also, far more than the 0.55 and 0.73% (72 and 96 26 bp tags, respectively) showing at least 8-fold down- or up-regulation in nodules of the same plants. With the highest up-regulation level, a 26 bp tag annotated to a putative basic PR1 precursor (Q3LF77_PEA) was highly induced and most differentially expressed (R_ln _= 4.34, >70-fold induced). An early nodulin class 40 (Enod40, NO40_SESRO) showed the second highest induction level. This is the first report of a dramatic induction of an Enod40 gene in legumes under salt-stress. Apart from its function in the early stages of nodule formation, Enod40s may also modulate the action of auxin, and function as plant growth regulators altering phytohormone responses (http://www.uniprot.org/uniprot/O24369)[25].

**Figure 3:**
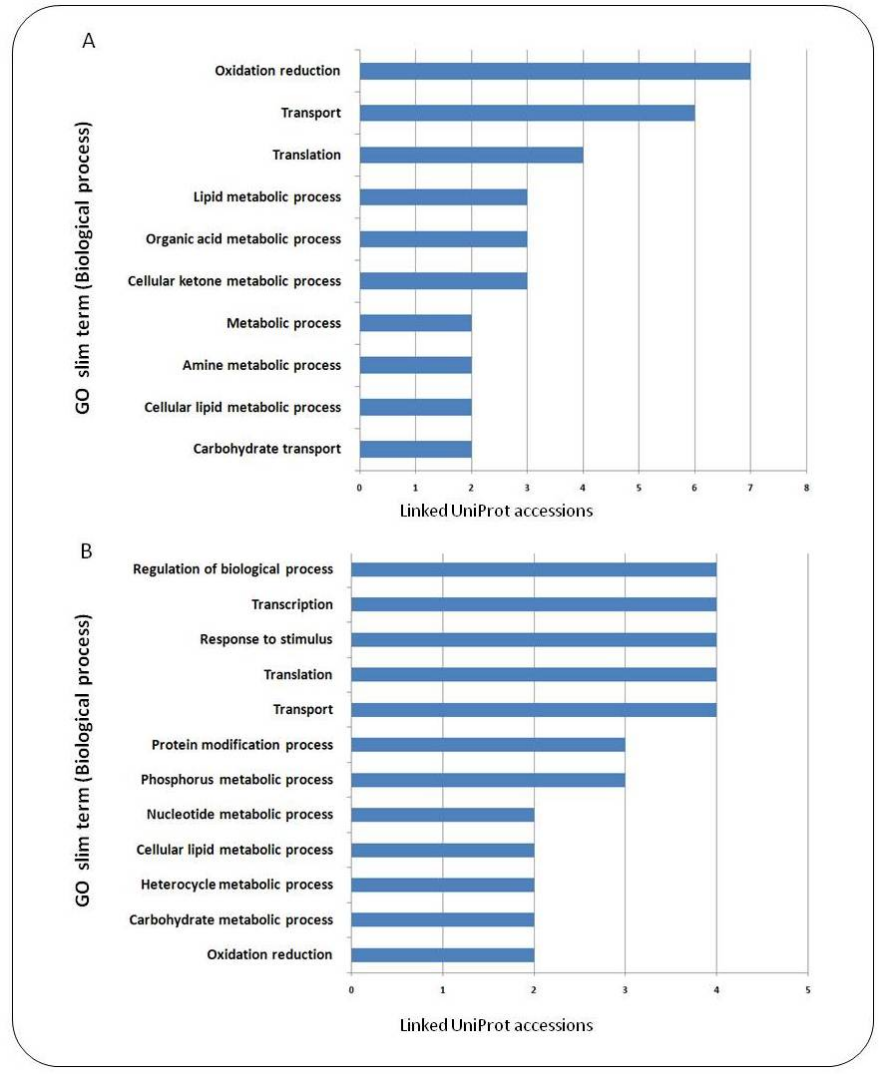
**Representation of GO slim terms among the 40 most up-regulated UniTags in salt-stressed roots and nodules**. A) Most represented GO slim terms for the most up-regulated UniTags in salt-stressed roots. B) Most represented GO slim terms for the most up-regulated UniTags in salt-stressed nodules.

The top 40 salt stress up-regulated transcripts from chickpea roots are deposited in Table 1. GO slim (biological process) statistics for the corresponding UniProt accessions are depicted in Figure 3.

**Table 1:** Top 40 salt stress up-regulated annotatable UniTags from INRAT-93 roots

Tag ID	Protein	Fold change	Uniprot ID
STCa-16261	Putative basic PR1 precursor	77,09	Q3LF77_PEA
STCa-18884	Early nodulin	60,95	NO40_SESRO
STCa-19168	Lipoxygenase	49,50	Q43817_PEA
STCa-7896	Superoxide dismutase	40,57	Q9ZNQ4_CICAR
STCa-318	Trypsin protein inhibitor 3	36,13	Q5WM51_CICAR
STCa-5894	General substrate transporter	34,09	A2Q5Z1_MEDTR
STCa-21968	Aquaporin	34,09	Q8W4T8_MEDTR
STCa-5877	Alternative oxidase	30,85	Q84KA1_CROSA
STCa-19021	Extensin	30,02	O65760_CICAR
STCa-17087	Dormancy-associated protein	29,22	O22611_PEA
STCa-283	Plastid phosphate translocator	27,58	A3RLB0_VICNA
STCa-7166	Isocitrate dehydrogenase (NADP)	25,97	IDHP_MEDSA
STCa-10582	Chalcone reductase	25,97	Q40310_MEDSA
STCa-6410	Predicted protein	25,56	A9V7Z1_MONBE
STCa-24417	Lipoxygenase	24,34	B7Z177_PEA
STCa-1381	Acetyl CoA synthetase	24,34	Q8LPV1_DESAN
STCa-2982	Cysteine synthase	23,52	Q8W1A0_SOYBN
STCa-24330	ABC transporter	21,91	O28298_ARCFU
STCa-20215	Putative extracellular dermal glycoprotein	21,91	Q9FSZ9_CICAR
STCa-13750	Glucose/galactose transporter	21,09	Q87CB9_XYLFT
STCa-22299	Predicted protein	20,70	A9TXV0_PHYPA
STCa-21916	Mob1-like protein	20,70	Q2WBN3_MEDFA
STCa-20066	14-3-3-like protein	20,70	A5YM78_CICAR
STCa-18427	Ribosomal Protein 117	20,29	Q7X9K1_WHEAT
STCa-24398	40S ribosomal protein S25	20,29	RS25_SOLLC
STCa-23821	ADP-ribosylation factor	20,29	Q6S4R7_MEDSA
STCa-1885	Mob1-like protein	19,47	Q2WBN3_MEDFA
STCa-387	CPRD49 protein	19,47	Q9AYM5_VIGUN
STCa-22950	Rubber elongation factor	19,47	Q2HUF4_MEDTR
STCa-21993	Isoliquiritigenin 2'-O-methyltransferase	19,47	CHOMT_MEDSA
STCa-17434	Chitinase-related agglutinin	18,67	A1YZD2_ROBPS
STCa-20130	Pectinesterase	17,85	Q2HRX3_MEDTR
STCa-23784	Predicted protein	17,85	Q2GRI0_CHAGB
STCa-4531	Cytochrome P450 monooxygenase	17,85	Q9SML1_CICAR
STCa-22619	Predicted protein	17,85	A9SIK2_PHYPA
STCa-4616	60S ribosomal protein	17,05	Q84U89_MEDSA
STCa-10115	Cytochrome b561	17,05	A2Q4A8_MEDTR
STCa-12309	Probable methyltransferase	17,05	PMTQ_ARATH
STCa-1385	1-aminocyclopropane-1-carboxylate oxidase	17,05	Q9XER2_TRIRP
STCa-14437	60S acidic ribosomal protein P1	17,05	RLA1_MAIZE

Among the GO slim terms (biological processes) linked to the most up-regulated root UniTags, oxidation-reduction occupied the highest rank, being represented by transcripts annotated to Q9ZNQ4_CICAR (Superoxide dismutase), Q9XER2_TRIRP (1-aminocyclopropane-1-carboxylate oxidase), Q9SML1_CICAR (Cytochrome P450 monooxygenase), B7Z177_PEA and Q43817_PEA (Lipoxygenases), Q84KA1_CROSA (Alternative oxidase), and Q40310_MEDSA (Chalcone reductase). Although not directly oxidation-reduction related, cellular ketone metabolic process was added to this group through association with lipoxygenases reported above and the accession Q8W1A0_SOYBN (Cysteine synthase). Further on, the translation biological process was represented by A9TXV0_PHYPA (predicted protein), Q84U89_MEDSA (60S ribosomal protein), Q7X9K1_WHEAT (Ribosomal Pr 117), and RLA1_MAIZE (60S acidic ribosomal protein). These results suggest a strong activation of ROS-scavenging mechanisms, a very well known event in stressed plant tissues, and deploy of the protein machinery as prime responses in the stressed roots. However, information based on only the top 40 up-regulated UniTags should not be considered as representative for the whole transcriptome. In subsequent sections, representation-analysis of GO terms, that take the expression level of the complete set of annotated UniTags into account, will be assessed.

Simultaneously with the analysis of whole-transcriptome responses to salt stress in roots, nodules of the same plants were separately harvested for the establishment of SuperSAGE libraries (control and 2 h 25 mM NaCl-treatment, respectively). In contrast to salt-stressed chickpea roots (346 UniTags up-, 2055 down-regulated), only 95 and 72 UniTags, respectively, were at least 8.0-fold up- or down-regulated. The top 40 most up-regulated transcripts in chickpea nodules after 2 hours of salt stress are listed in Table 2. GO slim (biological process) statistics for the corresponding UniProt accessions are depicted in Figure 3.

**Table 2:** Top 40 up-regulated annotatable UniTags in salt stressed nodules

Tag ID	Protein	Fold induction	Uniprot ID
STCa-18884	Early Nodulin	61,44	NO40_SESRO
STCa-11090	40S ribosomal protein SA	15,36	RSSA_CICAR
STCa-5362	18.2 kDa class I heat shock protein	13,65	HSP12_MEDSA
STCa-22299	Predicted protein	13,65	A9TXV0_PHYPA
STCa-17434	Chitinase-related agglutinin (Fragment)	13,65	A1YZD2_ROBPS
STCa-2116	Syringolide-induced protein B13-1-9	13,65	Q8S8Z8_SOYBN
STCa-9450	ATPase 9	13,65	PMA9_ARATH
STCa-13463	Formin I2I isoform	13,65	Q8H1H2_SOLLC
STCa-24417	Lipoxygenase	12,79	B7Z177_PEA
STCa-5357	Phosphatidylinositol transfer-like protein III	11,94	Q94FN1_LOTJA
STCa-89	Cold-induced protein	11,94	Q6PNN7_9FABA
STCa-15605	BRI1-KD interacting protein 109	11,94	Q762A5_ORYSJ
STCa-8350	Isopentenyl pyrophosphate isomerase	11,94	Q6EJD1_PUELO
STCa-5037	Phytochrome A	11,94	PHYA_PEA
STCa-175	Transcription elongation factor 1 homolog	10,24	ELOF1_ARATH
STCa-7855	Abnormal suspensor SUS2	10,24	UPI000016331D
STCa-705	MAP kinase protein	10,24	Q9SMJ7_CICAR
STCa-2196	SRC2	10,24	O04133_SOYBN
STCa-6099	Pyruvate kinase	10,24	Q5F2M7_SOYBN
STCa-305	FAM10 family protein	10,24	F10AL_ARATH
STCa-10862	Os04g0591100 protein	10,24	Q0JAL2_ORYSJ
STCa-933	Major histocompatibility class I receptor	10,24	Q95I97_9PERO
STCa-13055	Non-specific lipid-transfer protein precursor	10,24	NLTP_CICAR
STCa-6059	Protein RIK	10,24	RIK_ARATH
STCa-15235	L3 Ribosomal protein	10,24	Q9SBR8_MEDVA
STCa-20520	Elongation factor 1-alpha	10,24	Q3LUM5_GOSHI
STCa-11119	Fiber annexin	10,24	O82090_GOSHI
STCa-1896	Protein kinase-like protein	9,38	Q56YK2_ARATH
STCa-19301	F-box/kelch-repeat protein	8,53	FBK22_ARATH
STCa-5894	General substrate transporter	8,53	A2Q5Z1_MEDTR
STCa-5877	Alternative oxidase	8,53	Q84KA1_CROSA
STCa-1885	Mob1-like protein	8,53	Q2WBN3_MEDFA
STCa-170	Mob1-like protein	8,53	Q2WBN3_MEDFA
STCa-16125	Cytochrome c oxidase subunit 6b	8,53	Q8LD51_ARATH
STCa-8434	Fiber protein Fb2	8,53	Q8GT87_GOSBA
STCa-18178	Histone H2A.2	8,53	H2A2_MEDTR
STCa-2067	NAC family transcription factor 4	8,53	C7AFG1_CICAR
STCa-9977	T1K7.26 protein	8,53	Q9FZC2_ARATH
STCa-4833	WRKY27	8,53	Q2PJR9_SOYBN
STCa-11765	Putative uncharacterized protein	8,53	A2Q3F3_MEDTR

In comparison to roots of the same plants, highly expressed nodule transcripts originated from genes encoding TFs and transport-related proteins. Among the represented GO slim biological process in the most 40 up-regulated UniTags in salt stressed nodules (Figure 3), transcription and regulation of biological processes were represented by the UniProt entries ELOF1_ARATH (Transcript elongation factor 1), C7AFG1_CICAR (NAC family transcription factor 4), Q2PJR9_SOYBN (WRKY27 transcription factor), and PHYA_PEA (Phytochrome A). The response to stimulus process was also represented by accessions like F10AL_ARATH (FAM10 family protein) and HSP12_MEDSA (18.2 kDa class I heat shock protein). Further on, translation process was in turn represented by A9TXV0_PHYPA (predicted protein), and Q3LUM5_GOSHI (Elongation factor 1-alpha), whereas transport was represented by Q94FN1_LOTJA (Phosphatidylinositol transfer-like protein III), PMA9_ARATH (ATPase 9), NLTP_CICAR (Non-specific lipid-transfer protein precursor), A2Q5Z1_MEDTR (General substrate transporter), and Q762A5_ORYSJ (BRI1-KD interacting protein 109).

In addition to the expression profiles under stress conditions, differentially expressed UniTags in non-stressed nodules versus non-stressed roots from the same INRAT-93 plants were as well detected. A total of 51,545 tags from both untreated libraries represented 11,525 different UniTags. A total of 7,941 UniTags showed >3.0-fold differential expression between both organs. Of these, 2,098 UniTags with >3.0-fold differential expression were more prevalent in nodules. With a higher threshold, 140 transcripts were more than 8.0-fold prevalent in the symbiotic organs. All organ- and stress-related differentially expressed UniTags along with their respective annotations are deposited in Additional file 1.

**Figure 4:**
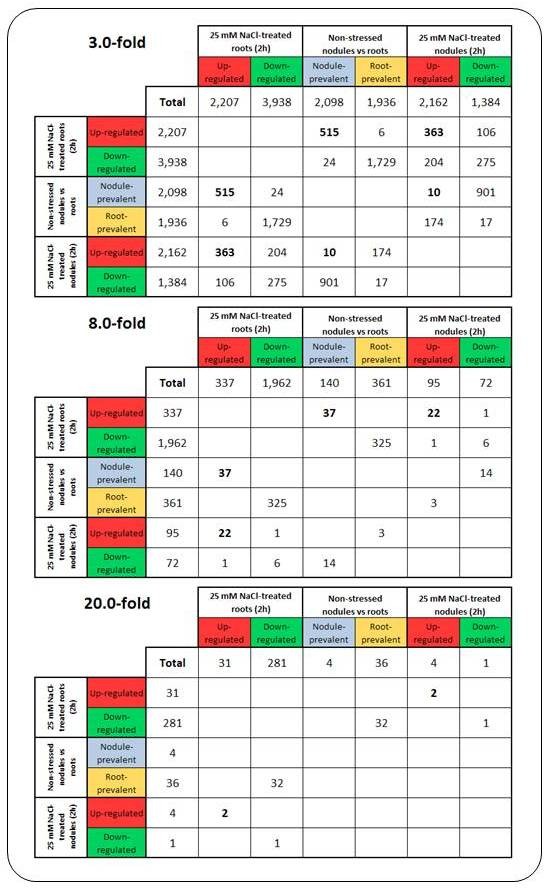
**Venn Mapper output detailing shared responses (number of UniTags) between salt-stressed roots and nodules, respectively, and non-stressed nodules relative to roots**.

Setting a minimum threshold of 3-fold differential expression, from the 2,098 26 bp tags prevalent in non-stressed nodules, 515 (24.5%) were also at least 3-fold up-regulated in roots under salt stress. These 515 UniTags represented 23.3% of the root transcripts >3-fold up-regulated by salt. On the other hand, only 10 out of the 2,098 UniTags were more than 3-fold up-regulated in salt-stressed nodules. In both salt-stressed roots and nodules, 363 common 26 bp tags were more than 3-fold up-regulated (16.7% from nodules, and 16.4% from roots; Figure 4, upper panel). As far as down-regulation is concerned, 1,729 out of 1,936 UniTags prevalent in non-stressed roots were more than 3-fold down-regulated in roots after 2 h of salt treatment. A total of 275 tags were commonly more than 3-fold down-regulated in both roots and nodules under salt-stress.

If the threshold is set to 8-fold differential expression, 37 out of 140 tags prevalent in non-stressed nodules were more than 8-fold up-regulated in salt-stressed roots. Upon salt stress in both organs, 22 UniTags were commonly more than 8-fold up-regulated. On the other hand, no tags prevalent in non-stressed nodules were more than 8-fold up-regulated in the same organ upon salt stress (Figure 4, mid panel).

Four 26 bp tags were more than 20-fold differentially expressed between non-stressed nodules and roots. From these, no shared response with any >20-fold salt-induced root or nodule transcript was observed (Figure 4, lower panel). Annotatable tags shared by salt-stressed nodules and roots with a minimum threshold of 8-fold up-regulation in response to salt stress are listed in Table 3.

**Table 3:** Overlapping transcriptome responses of UniTags that are highly up-regulated in roots and nodules upon salt stress

Tag ID	Protein	Fold induction (roots)	Fold induction (nodules)	Associated process	Uniprot ID
STCa-18884	Early nodulin	60,95	61,44	Nodulation	NO40_SESRO
STCa-22299	Predicted protein	20,70	13,65	Unknown	A9TXV0_PHYPA
STCa-17434	Chitinase-related agglutinin	18,67	13,65	Defense	A1YZD2_ROBPS
STCa-24417	Lipoxygenase	24,34	12,79	Lypid metabolism	B7Z177_PEA
STCa-5357	Phosphatidylinositol transfer-like protein III	8,12	11,94	Unknown	Q94FN1_LOTJA
STCa-175	Transcription elongation factor 1	13,79	10,24	Transcription	ELOF1_ARATH
STCa-7855	Abnormal suspensor SUS2	9,74	10,24	Unknown	UPI000016331D
STCa-705	MAP kinase protein	8,12	10,24	Signal transduction	Q9SMJ7_CICAR
STCa-5894	General substrate transporter	34,09	8,53	Transport	A2Q5Z1_MEDTR
STCa-5877	Alternative oxidase	30,85	8,53	Redox homeostasis	Q84KA1_CROSA
STCa-1885	Mob1-like protein	19,47	8,53	Cytokinesis	Q2WBN3_MEDFA
STCa-170	Mob1-like protein	12,17	8,53	Cytokinesis	Q2WBN3_MEDFA
STCa-16125	Cytochrome c oxidase subunit 6b	11,36	8,53	Redox homeostasis	Q8LD51_ARATH
STCa-8434	Fiber protein Fb2	10,55	8,53	Unknown	Q8GT87_GOSBA
STCa-18178	Probable histone H2A.2	9,74	8,53	DNA stability	H2A2_MEDTR
STCa-15648	GR395157.1 *C.arietinum *EST	22,30	23,87	Uncharacterized	-
STCa-19240	GR916593.1 *C.arietinum *EST	10,54	10,23	Uncharacterized	-
STCa-11740	GR408054.1 *C.arietinum *EST	10,54	11,94	Uncharacterized	-
STCa-15130	Contig45449 *C.arietinum *EST	8,11	8,53	Uncharacterized	-
STCa-7445	Contig739 *C.arietinum *EST	17,84	10,23	Uncharacterized	-
STCa-1958	No homologous EST	14,61	13,65	Un-annotated	-
STCa-8135	No homologous EST	8,11	8.53	Un-annotated	-

Threshold in organ-wise differential expression: R_(ln) _2.0 (8-fold)

Threshold in stress-treatment differential expression: R_(ln) _2.0 (8-fold)

### Global transcriptome differences in stressed roots and nodules

To uncover global differences between the root and nodule transcriptomes, Gene Score Re-sampling analysis (GSR) for over-representation of GO functional categories was carried out for the total annotated UniTags from both organs. The GSR analysis was fed with the expression levels (fold changes) of each UniTag after stress induction. The GO categories (biological process) undergoing the most significant changes in a stress- and organ-manner were then detected.

Additional to standard GO terms, two custom-made categories were introduced to the GSR analysis, following the procedure of Gillis and co-workers [26]. Over-representation within our data set from transcripts involved in the salt overly sensitive pathway (SOS), and ROS-scavenging mechanisms (SODs, ascorbate and glutathione cycles) was in this way confirmed. Genes belonging to both pathways were filtered from already enriched GO terms and were re-entered into the analysis. A closer detail on both aspects will be assessed on the discussion section.

**Figure 5:**
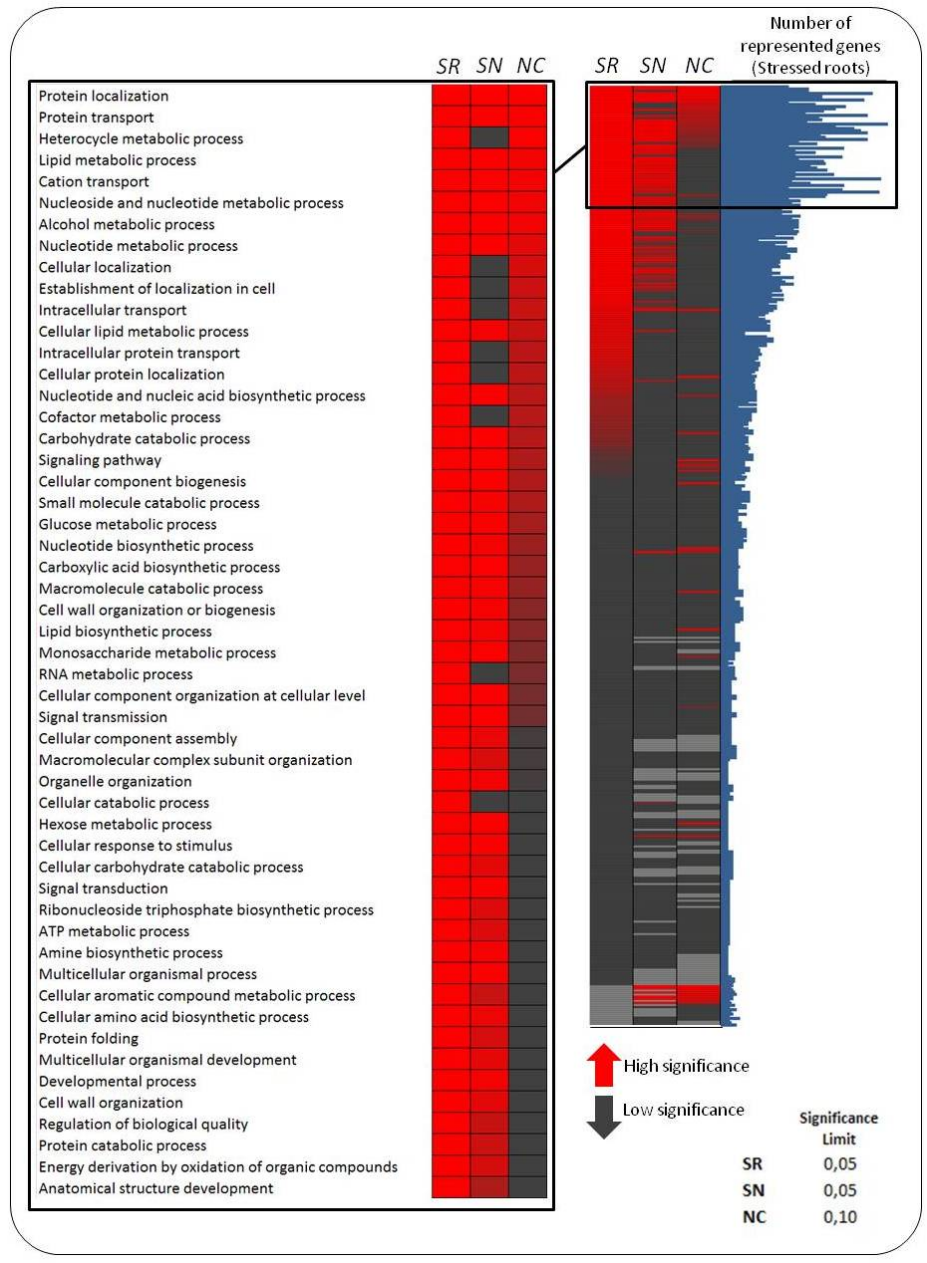
**GSR analysis of over-represented GO biological terms in salt-stressed roots and nodules**. Right panel: Graphic representation of significant over-represented GO biological processes in stressed roots (SR), stressed nodules (SN), and non-stressed nodules versus non-stressed roots (NC). Numbers of represented genes per GO category in salt stressed roots are represented by the blue bars next to the heat map. Left Panel: Detail showing the 52 most over-represented biological processes for salt stressed roots, compared to stressed and control nodules.

In chickpea roots from control and salt-treated libraries, a total of 6,637 UniTags were associated to Gene Ontology (GO) terms. After GSR analysis of 450 biological processes, a total of 191 terms were over-represented with (P < 0.05). From them, 52 biological processes were over-represented with very high significance (P < 5E-10). A graphical overview of the GSR results for salt stressed chickpea roots, along with the most significant over-represented GO categories and their representation in nodules of the same plants is depicted in Figure 5.

Not surprisingly, signal-related processes like GO terms GO:0023033 (Signalling pathway), GO:0007165 (Signal transduction), and GO:0023060 (Signal transmission) were over represented. Transport categories like GO:0015031 (Protein transport) and GO:0006812 (Cation transport) were also highly significant. Among the metabolism-related processes, lipid metabolism-related categories (GO:0006629, GO:0044255, GO:0008610) interestingly reacted upon salt stress. Concerning protein homeostasis, terms related to transport (GO:0015031), folding (GO:0006457), localization (GO:0008104), and catabolism (GO:0030163) also showed P < 1E-10 values.

Diverse response-related terms with different significance levels were also detected. As an example, GO term GO:0051716 (Cellular response to stimulus) was represented by 60 UniProt accessions, showing highly significant over-representation (P < 1E-10), whereas GO term GO:0033554 (Cellular response to stress) showed a lower significance level (P < 1E-4). Globally, from 31 response-related terms, 9 were over-represented with at least P < 1E-3. Additionally, quite as expected, SOS- and ROS-custom categories resulted over-represented with (P < 0.05)

Nodules of the same INRAT-93 plants differed from roots in their global transcriptome remodelling level after stress induction. From the 450 analysable terms, 102 GO terms were over represented with (P < 0.05). With higher significance levels, 15 and 26 biological processes showed P values of (P < 5E-10) and (P < 5E-4), respectively. Ten of the GO terms with very high significance (P < 5E-10) were also over-represented in roots of the same plants. Depending on the organ, some sets of different UniProt accessions were associated with each GO term. A clear example is the GO term GO:0006629 (Lipid metabolic process). In roots, 91 UniProt accessions were associated to this term, whereas only 68 were associated in nodules. From them, 6 were not common to both organs (but only associated in nodules). Another example is GO:0009117 (Nucleotide metabolic process), which is represented by 75 and 65 UniProt accessions in roots and nodules, respectively. From them, 16 showed either nodule- or root-specific association.

Concerning processes more induced in stressed nodules while showing no significant representation level in roots, the terms GO:0006508 (Proteolysis), GO:0006811 (Ion transport), GO:0042221 (Response to chemical stimulus), GO:0044106 (Cellular amine metabolic process), and GO:0006022 (Aminoglycan metabolic process) revealed the highest contrasts (Figure 5). On the other hand, 35 terms were more represented in stressed roots, among them, the SOS-custom category). From them, 12 terms showed (P < 1E-10) and (P > 0.5) over-representation significances in stressed roots and nodules, respectively (Figure 5). A complete matrix with all analysed processes along with their significance levels of over-representation, and the associated UniProt entries for the different organs and stress conditions are deposited in Additional file 2.

### Nodule over-represented GO biological processes under control conditions

To reveal global transcriptome differences in both organs under control conditions, the differential expression of UniTags from untreated nodules compared to roots was calculated, and the fold changes were fed into GSR analysis. In non-stressed tissues, the levels of significance for over-representation estimated by the ErmineJ package were substantially lower compared to the ones observed in salt stressed roots and nodules. In control conditions, a total of 15 and 72 GO biological processes were more prevalent in nodules with (P < 0.05) and (P < 0.1), respectively (Additional file 2).

Interestingly, 33 of the GO biological processes prevalent in nodules (P < 0.1) were over-represented in salt stressed roots at very high significance levels (P < 1E-10). Protein machinery- (GO:0008104, GO:0015031, GO:0006886), lipid metabolism- (GO:0006629, GO:0044255), and general metabolism-related (GO:0006066, GO:0051186, GO:0044282, GO:0006006) terms were among these common processes. From 15 GO terms prevalent in nodules with (P < 0.05), 10 were over-represented in salt-stressed nodules with the same significance threshold. Among these common processes, highest on the rank of over-representation in control nodules, the ROS custom category showed a significance of (P < 0.013). All common over-represented GO biological processes in untreated nodules and salt-stressed roots are summarized in Table 4.

**Table 4:** Commonly over-represented GO biological processes in chickpea salt stressed roots and non-treated nodules

GO Biological process	GO Term ID	P value (NaCl Roots)	P value (ctr nodules)
Protein transport	GO:0015031	8,76E-12	0,0235
Protein localization	GO:0008104	9,98E-12	0,0293
Alcohol metabolic process	GO:0006066	2,04E-11	0,0391
Nucleoside and nucleotide metabolic process	GO:0055086	6,13E-11	0,0424
Cation transport	GO:0006812	2,15E-11	0,0427
Lipid metabolic process	GO:0006629	1,07E-10	0,0469
Heterocycle metabolic process	GO:0046483	2,38E-11	0,0587
Nucleotide metabolic process	GO:0009117	4,29E-11	0,0621
Cellular localization	GO:0051641	8,58E-12	0,0646
Cellular lipid metabolic process	GO:0044255	1,05E-11	0,0668
Small molecule catabolic process	GO:0044282	1,19E-11	0,0698
Establishment of localization in cell	GO:0051649	8,41E-12	0,0710
Intracellular transport	GO:0046907	1,13E-11	0,0712
Cellular component biogenesis	GO:0044085	8,58E-11	0,0715
Nucleotide and nucleic acid biosynthetic process	GO:0034654	1,79E-11	0,0726
Cellular protein localization	GO:0034613	1,26E-11	0,0736
Cofactor metabolic process	GO:0051186	7,15E-11	0,0739
Carbohydrate catabolic process	GO:0016052	1,23E-11	0,0740
Signaling pathway	GO:0023033	1,95E-11	0,0751
Glucose metabolic process	GO:0006006	1,16E-11	0,0755
Intracellular protein transport	GO:0006886	1,65E-11	0,0758
Nucleotide biosynthetic process	GO:0009165	1,38E-11	0,0766
Macromolecule catabolic process	GO:0009057	1,87E-11	0,0766
Carboxylic acid biosynthetic process	GO:0046394	1,72E-11	0,0774
RNA metabolic process	GO:0016070	1,48E-11	0,0829
Monosaccharide metabolic process	GO:0005996	2,26E-11	0,0831
Cellular component organization at cellular level	GO:0071842	8,94E-12	0,0842
Cell wall organization or biogenesis	GO:0071554	9,53E-12	0,0842
Lipid biosynthetic process	GO:0008610	3,58E-11	0,0859
Signal transmission	GO:0023060	3,06E-11	0,0860
Organelle organization	GO:0006996	1,59E-11	0,0958
Macromolecular complex subunit organization	GO:0043933	1,07E-11	0,0972
Cellular component assembly	GO:0022607	1,34E-11	0,0986
**ROS-scavengers**	**Custom**	**0,0088**	**0,0131**

### Information transfer from UniTag profiles to other platforms

#### *In situ *visualization of two ascorbate peroxidase (APX) transcripts in chickpea nodules

**Figure 6:**
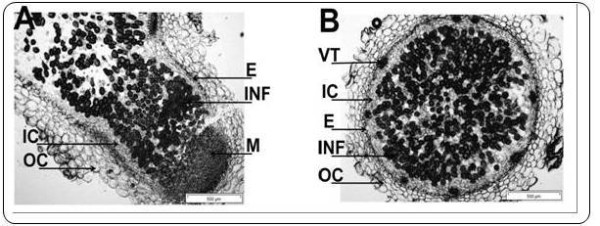
**Longitudinal (A) and cross section (B) through a chickpea nodule**. Abbreviations: (INF) Infected zone, (NP) Nodule parenchyma, (E) Endodermis, (NC) Nodule cortex, (VT) Vascular trace, (IC) Inner cortex, (OC) Outer cortex, (M) Meristem

To test the transferability of information between UniTag profiles and other platforms, selected nodule-metabolism-associated transcripts were localized in chickpea nodules. For this purpose, *in situ *PCRs of ascorbate peroxidase-annotated UniTags were performed in nodule slices. Main features of the anatomy of chickpea nodules are depicted in Figure 6.

**Figure 7:**
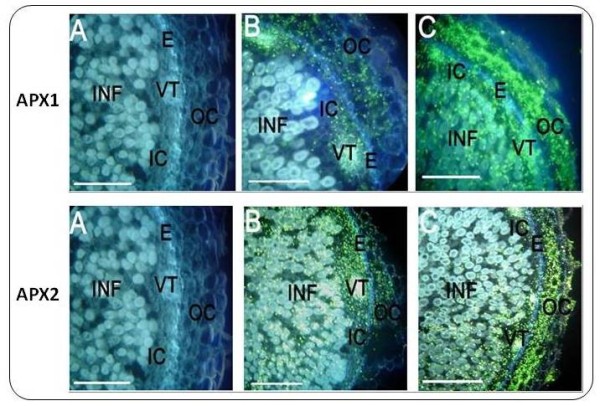
***In situ *localization of ascorbate peroxidase APX transcript isoforms in chickpea root nodules**. *In situ *localization of APX1 (upper panel) and APX2 (lower panel, bar: 500 μm). Corresponding cDNAs were derived from RACE amplifications using UniTags as starting sequences. A) Control without reverse transcription B) Nodules from non-stressed plants C) Nodules from plants after a 2 h exposure to 25 mM NaCl Abbreviations: see figure 6

Transcripts from the ascorbate peroxydase 1 (APX1) gene were detected mostly in the inner cortex and less in the outer cortex of control nodules (non-exposed to salinity) (Figure 6). Two hours after exposure to 25 mM NaCl, the transcripts accumulated to high levels in the outer cortex (Figure 7). Note, that the negative controls did not show any fluorescent signal. In contrast, only few transcripts from the APX2 gene appear in control root nodules, whereas the number of transcripts highly increased both in the inner and the outer cortex after exposure to salinity (Figure 7).

Ascorbate peroxidase (APX) is a key enzyme that scavenges potentially harmful H_2_O_2 _and thus prevents oxidative damage especially in N_2_-fixing legume root nodules. In fact, nodules have a high capacity to generate activated forms of O_2 _such as H_2_O_2_, because of the relatively high rates of respiration during the early response to moderate salt stress. The elevated levels of transcripts encoding ROS scavengers in the outer and inner cortex, like APX2 in our study, point to a critical role of these regions in ROS-scavenging, thereby counteracting by-products of respiration caused by leakage of the electron transport chain. Therefore, elevated levels of APX would be required to provide adequate antioxidant defence. Although nodules possess an array of antioxidant metabolites and enzymes that prevent the generation of highly oxidizing radicals (as e.g. O^2-^, OH^.^) and hence the damage of lipids, proteins, and DNA, to name some. In addition, antioxidants regulate the intracellular concentrations of reactive oxygen species, such as the superoxide radical and hydrogen peroxide (H_2_O_2_), that are signalling stress perception and activating stress-responsive genes [27,28].

#### Quantification of oxylipin-related UniTags via qPCR

The GSR analysis in salt-stressed chickpea roots and nodules revealed lipid-metabolism associated categories among the stress over-represented GO biological processes (GO:0006629, GO:0044255, GO:0008610). Lipoxygenases and other oxylipin-related UniProt accessions belong to genes (proteins) most frequently associated with the respective GO terms (http://www.ebi.ac.uk/QuickGO/GTerm?id=GO:0006629).

Oxidized fatty acids, known as oxylipins, are compounds tightly linked to ROS metabolism and stress responses in plants [29]. These compounds, which are generated by the coordinated action of lipases, lipoxygenases, and some cytochrome P450 enzymes, are key elements of jasmonic acid (JA) biosynthesis. JA represents a signalling molecule, that, apart from stress responses, is also involved in developmental processes [30]. A set of oxylipin-related transcripts was selected and confirmed by qRT-PCR, and their expression levels were compared in different chickpea varieties. Among more than 20,000 surveyed transcripts, 20 UniTags annotated to lipoxygenases were distributed over 11 SAATs families and annotated to 9 different UniProt accessions. These UniTags varied their expression levels from 5fold down-(STCa-20253) to 25fold up-regulation (STCa-24417) 2 h after onset of salt stress in chickpea roots.

Allene oxide cyclase (AOS) is an enzyme involved in JA biosynthesis by catalysing the conversion of fatty acid hydroxyperoxides to 12-oxophytodienoic acid (OPDA) [31]. In chickpea roots and nodules, five UniTags could be annotated to two AOS UniProt accessions. Expression levels for the respective UniTags varied from 4-fold up-regulation to 20-fold down regulation.

**Figure 8:**
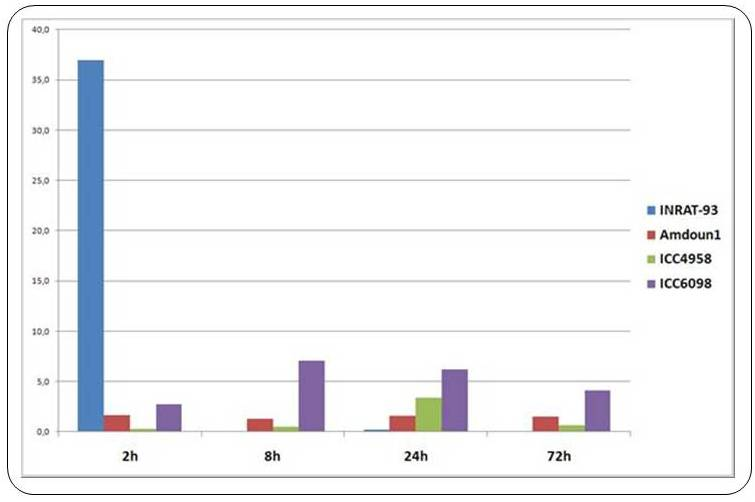
**Relative expression levels of AOS-annotated transcripts in four chickpea varieties at different time points after salt stress onset, measured by qRT-PCR**. The expression level of the INRA-93 derived 26 bp tag STCa-13267, annotated to an allene oxide synthase (AOS), was determined in the salt-tolerant chickpea variety (INRAT-93) and compared to less tolerant varieties. According to deepSuperSAGE, the AOS-annotated transcript is slightly (1,6-fold) down-regulated 2 h after stress, but the relative expression level remains very high when compared to the one of the salt-sensitive varieties. At subsequent time points, the AOS-expression decreases drastically in INRAT-93, whereas the levels in the salt-sensitive varieties remain constant.

Comparative qRT-PCR assays using RNAs from chickpea varieties INRAT-93 (salt-tolerant), Amdoun1 (salt-sensitive), ICC-4958 (sensitive), and ICC-6098 (weakly tolerant) confirmed the deepSuperSAGE data of selected UniTags 2 h after salt stress in INRAT-93. Additionally, marked differences in expression levels were detected as compared to other varieties with lower salt tolerance. A good example is the AOS-annotated transcript STCa-13267 (1,6-fold down-regulated in INRAT-93). After comparing the expression of the same AOS transcript via qRT-PCR (using STCa-13267 as starting sequence) in INRAT-93, ICC-4958, and ICC-6098, much higher expression levels were detected in INRAT-93 (especially 2 h after stress onset, figure 8).

**Figure 9:**
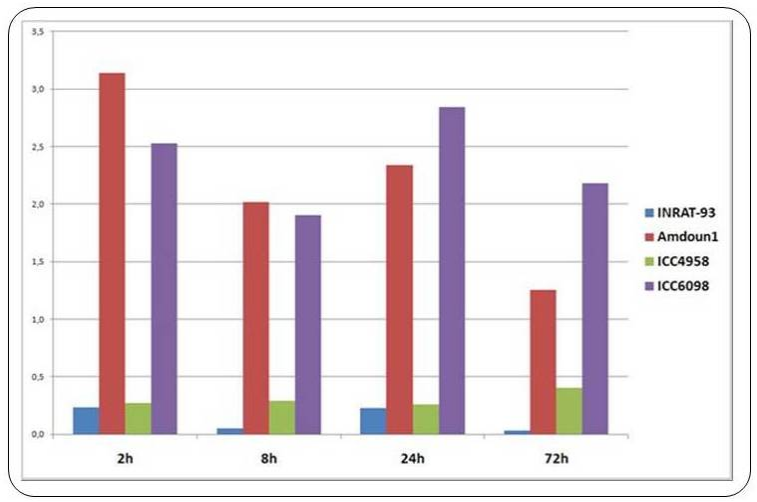
**Relative expression levels of a lipoxygenase-annotated transcript in four chickpea varieties at different time points after salt stress onset, measured by qRT-PCR**. Transcript levels of chickpea 26 bp tag STCa-7252, annotated to a lipoxygenase gene, remained constant 2 h hours after salt stress onset in INRAT-93 according to deepSuperSAGE. When compared to the transcript levels of other chickpea varieties, strong differences are observed. The two less salt-tolerant varieties Amdoun1 and ICC6098 show a much higher expression level over time.

Concerning up-regulated transcripts of the salt-tolerant variety INRAT-93, cases in which the level of up-regulation was still higher in a salt-sensitive variety were also observed. As an example, the lipoxygenase-annotated 26 bp tag STCa-7252, which is 2,5-fold up-regulated under salt stress in INRAT-93, is much stronger up-regulated in salt-sensitive Amdoun1. This suggests that some of the stress-related genes "over-react" in salt-sensitive plants, which may account for the differences between salt-tolerant and -sensitive varieties (Figure 9). SuperSAGE and qRT-PCR expression profiles of the selected genes in the tested varieties are deposited in Additional file 1.

#### Confirmation of SuperSAGE expression profiles by microarray hybridization

SuperSAGE libraries were developed from chickpea plants exposed to other abiotic stresses such as cold (data not shown) and drought stress [32]. From these, a selection of approximately 3,000 26 bp tags with diverse regulation levels were spotted onto an Agilent™ 16K micro-array (AGILENT TECHNOLOGIES, Santa Clara CA, USA) as 26 bp oligonucleotides (1,200 spotted in duplicate). For each spotted 26 bp tag, similar oligos carrying mismatches were additionally spotted onto the array in three sets as follows: i) mismatch at position 7; ii) mismatches at positions 7 and 13, respectively; and iii) mismatches at positions 7, 13, and 20, respectively. Microarray design followed a previous report [33]. After background correction using the intensities of the oligos carrying mismatches, dye swapping, and statistical treatment, the expression ratios [R_(ln)_] from a selection of 660 26 bp oligos were compared with the original deepSuperSAGE profiles. Although any comparison of such different profiling platforms is hampered by the differences in signal magnitudes, the almost unavoidable cross-hybridization signals on micro-arrays, the sequence similarity between some spotted 26 bp tags with contrasting regulation levels, and the relatively short size of the spotted oligos (26 bp), a shared tendency towards up- or down-regulation of transcripts of 79.0% was observed between both platforms (See Additional file 1).

## Discussion

### Transcripts abundance and occurrence of SNPs-associated alternative tags

The tag copy numbers within the present chickpea deepSuperSAGE libraries reveal that a substantial proportion of the sampled transcripts is present at low abundance (at least 80% are found at 1 to 10 copies × 100,000^-1^). This observation is not at all new for plants. In SAGE-based transcriptome analyses in *Arabidopsis *and maize, at least 70% of the detected transcripts (excluding singletons) were of low abundance [34,35]. In several other organisms outside the plant kingdom (i.e. yeast, mouse, and humans), large differences between abundant and rare transcripts have also been observed [36].

It is not clear why this proportion of low copy number transcripts is so big in many transcriptomes. It has been suggested that transcripts found in high abundance represent a limited number of house-keeping genes, whereas "rare" transcripts are derived from genes with more specialized functions [37]. In other cases, transcripts encoding proteins with the same function can also display very different ranges of copy numbers [38].

Chickpea root and nodule low copy number UniTags belong to diverse functional categories comprising signalling-, general metabolism-, transcription regulation-, and protein machinery-related biological processes (Additional file 1). Moreover, we observed that some low copy number UniTags accompany high copy number SAATs. Future studies can profit from the information generated in the present work, where more than 13,000 chickpea low copy number transcripts variants are potential candidates for further validation.

Concerning sequence similarity, more than 20,000 UniTags derived from chickpea roots and nodules were analyzed, permitting to follow the expression dynamics of equivalent 20,000 hypothetical complete transcripts. However, how redundant is the original mRNA population, and what level of variation is observed between transcripts, still remain questions to further explore. We have observed that around 70% of the transcripts contained in a certain chickpea library are different to each other, whereas the remaining 30% can form families of very similar 26 bp tags, many of them with contrasting expression profiles. Up to date, no extensive study of this phenomenon in plants exists, that could be exploited for the explanation of the observed results. However, in humans, as exemplarily reported for cancer cells, tags generated by SAGE-related techniques are very often differentiated from each other only by SNPs [39]. Extensive studies in which the tag-to-transcript assignment has been approached in deep resolution show that SNP-associated alternative tags (here named SAATs) were observed for nearly 8,6% of all known human genes. Additionally, from all analyzed transcripts, 2.6% harbored a SNP contiguous to a terminal *NlaIII *(the SuperSAGE tagging enzyme) recognition site [22].

Considering the appearance of very similar transcripts in an organism, the occurrence of SNPs within ESTs in humans has been associated to the high flexibility of the transcriptome that allows the generation of varying transcript "isoforms" from a single genomic locus, a phenomenon also reported in *Arabidopsis *[40-42]. In several cases these small sequence differences have been associated to changes in RNA stability and/or decay rate, translation rates, cell- and tissue-localization, and stability of the produced protein [43-45].

The present study delivers valuable information from more than 6,000 chickpea transcripts that present small sequence variations, and nevertheless very different expression levels under salt stress conditions. Future studies in which finer tissue- or cell type-resolution is achieved may profit from the present data, and answer several questions, that up to now remain open.

### Salt stress-induced differential gene expression in chickpea roots

After analysis of more than 17,500 26 bp tags from chickpea roots, UniTag STCa-16261 (Q3LF77_PEA, pathogenesis-related leaf protein 1, PR1) represented the most up-regulated transcript in salt-treated chickpea roots with a more than 70fold up-regulation. Proteins of this family have previously been associated to defense responses and plant stress (http://pir.georgetown.edu/cgi-bin/ipcSF?id=PIRSF002704). Apart from STCa-16261 (present in 184 copies × 100,000^-1^), 9 similar alternative UniTags are annotated to the same EST (all found in low copy numbers). Although it is known that the encoded proteins are most probably involved in extracellular signaling, no targets or activators are known. The present results expand our knowledge of PR1-related proteins and delivers already 10 transcript isoforms with different abundance levels.

Further down the list of top up-regulated UniTags, at least three UniProt accessions associated to ROS-scavenging and redox homeostasis are found, represented by UniTags annotated to one superoxide dismutase (Q9ZNQ4_CICAR, SOD), two lipoxyenases (Q43817_PEA, B7Z177_PEA), and one alternative oxidase (Q84KA1_CROSA). Reports on high activity of ROS-related genes under stress are already known from plants, an aspect that has been extensively reviewed elsewhere [46]. However, to extract transcript populations for candidates with pronounced stress responses is a crucial task. As an example, transcript profiles of at least further 27 lipoxygenase-annotated transcripts are reported here (Additional file 1).

Related to genes from other functional categories, Trypsin inhibitors rapidly accumulate in plants under salt, drought, high aluminum stress, wounding, fungal infection, and ABA and jasmonate applications [47]. Particularly in the first stages of salt stress response in rice, various trypsin inhibitor isoforms are very active [48]. Extensin proteins are generally involved in cell-wall modifications to counteract mechanical pressures arising from differences in water potential [49,50]. Although dormancy-associated proteins are salt stress-induced in *M. truncatula*, very little is known about their exact functions [51]. As growth-promoting phytohormones, auxins function in the regulation of root development in salt-stressed plants [52]. Therefore, the over-expression of UniTag STCa-17087 (dormancy-associated protein, O22611_PEA) in salt-treated INRAT-93 roots may be linked to auxin activity and root growth regulation. Reports on the activity of the NADP^+^-dependent isocitrate dehydrogenase (ICDH) in the facultative halophyte *Mesembryanthemum crystallinum *already highlighted the importance of this protein under salt stress [53]. However, contrasting the results for chickpea, the activity of ICDH increased in leaves, and decreased in roots in *M. crystallinum*. Acetyl-CoA synthetase (ACS), a key enzyme in acetate production, does not seem to be involved in the management of osmotic or ionic stress. Only one study is known, in which acetate production levels and the activities of ACS and aldehyde dehydrogenase (ALD) were monitored under hyperosmotic conditions [54]. Also, informations about the role of cysteine synthases in plants under salt stress are rather limited. However, apart from its general role in protein biosynthesis and as a sulfur donor, cysteine is one of the main components of the anti-oxidant glutathione (along with glutamate) [55]. Glutamate and ascorbate are the major redox buffers in plants, representing the ascorbate/glutathione cycle [9].

Any one-by-one listing of all the up- or down-regulated transcripts under certain conditions is not efficient when high-throughput technologies are used, where thousands of genes are monitored in parallel. To date, several approaches overcome the handling of big data masses to extract biological meaning. In the present work, the accumulated large information body has been filtered combining the output of GSR (GO categories over-representation) analysis with literature and metabolic pathways available in public domains. In subsequent sections, a few relevant biological processes will be dissected in some detail.

### Up regulated transcripts in salt stressed nodules and common nodule-root responses

In the present work, the transcription profile of more than 13,000 nodule UniTags has been deciphered. Several functional categories were represented among the most up regulated UniTags, from which a good portion was also up-regulated in salt-stressed roots of the same plants. Several of the underlying genes have been already identified as being stress-responsive in plants, and also in legumes, as is the case with genes encoding alternative oxidases (AOS), lipoxygenases, MAP kinases, cytochrome C oxidases [56-60], transcription elongation factors, and phosphatidylinositol transfer proteins [61,62]. All of them representing proteins involved in widely studied signalling and oxidative stress counteraction processes.

Other highly up-regulated, but less known genes deserve attention as potential candidates for further characterization. Examples are genes encoding agglutinins, proteins whose activity under osmotic stress is only known from few plant studies [63,64]. Annotated to proteins of this class, UniTag STCa-17434 (A1YZD2_ROBPS) was 13- and 18-fold up-regulated in salt stressed chickpea nodules and roots, respectively. Another case is the high salt tress induction of Mob1-annotated transcripts in chickpea roots and nodules. The involvement of Mob proteins in plant cytokinesis and growth has been studied in detail, yet no connection to stress responses has been reported [65]. In chickpea roots and nodules, Mob1-annotated UniTags STCa-1885 and STCa-170 are at least 8-fold up-regulated in both organs under salt stress, and both UniTags are accompanied by 13 SAATs, many of them showing contrasting expression profiles.

Even less characterized are plant fiber proteins like Fb2, from which only few functional data are available up to date (http://www.uniprot.org/uniprot/Q8GT87.html). Contrary to several other highly stress-induced transcripts in chickpea roots and nodules, Fb2 proteins are represented by only one single UniTag (STCa-8434), without any related SAAT. However, we could annotate further 15 UniTags to at least six other Fb classes.

As described above, the present results encompass sets of transcript variants derived from previously described genes together with their expression profiles and sequences (and derived similarities). Although a compilation of stress-regulated genes is not new, the present information on the dynamics of their transcript variants in chickpea roots and nodules merits attention.

Also, an important objective in high-throughput transcriptomics is the discovery of new genes. Among the highly stress up-regulated (>8-fold) UniTags from chickpea nodules and roots (detailed in Table 3), five UniTags were annotated to ESTs with no homology to any characterized plant mRNA or genic DNA sequences. These ESTs showed no significant BlastX complete hits to proteins deposited in the public domain. The respective UniTag- and complete chickpea target ESTs-sequences from unannotated transcripts are deposited in Additional file 3.

### Cross-feedback of GO representation analysis and abiotic stress-related pathways

Handling the large masses of data derived from high-throughput transcriptome studies implies extensive filtering of information. In the present study, GSR over-representation analysis of GO categories provided a view of the global transcriptome of chickpea roots and nodules and its remodelling under stress. However, instead of defining discrete pathways, GO terms group genes or proteins according to their associated biological process, cell components, or molecular functions (http://www.geneontology.org). So we re-screened the output of our GSR analysis to monitor the dynamics of transcripts associated with pathways represented in certain GO categories. Here, two stress-related processes are discussed in detail, i) the regulation of cellular Na^+ ^homeostasis, and ii) counteraction of reactive oxygen species (ROS). Although these two processes are widely studied in plants, a detailed search for potential pathway members, their transcript isoforms and their expression profiles, is still missing in several non-model crops [66].

#### Na^+ ^homeostasis and potential salt overly sensitive (SOS) members in chickpea

In plants, the salt-sensitive-overly (SOS) pathway partly manages the excess of Na^+ ^ions under saline stress conditions by the interplay of calcineurin B-like (SOS3/CBL4) Ca^2+ ^sensors with CBL-interacting protein kinases (SOS2/CIPK24), that regulate directly the activity of the SOS1 protein (Na^+^/H^+ ^antiporter), and indirectly, a broad array of proton pumps (mostly H^+^ATPases) [67-70]. Additionally to SOS1 to 3, SOS4 and SOS5 proteins have been identified to interact with the other SOS members. SOS4 encodes a pyridoxal (PL) kinase, whereas SOS5 encodes a polypeptide partly homologous to proteins of the arabinogalactan class (AGP) [71].

The SOS cascade has been initially studied in detail in *Arabidopsis *knock-out mutants, and later on also in other plants like rice, poplar, and several brassica species [72-75]. In salt stressed chickpea roots, after a cross-fed screening between the GSR results and SOS pathway members, four highly over-represented (P < 1E-10) biological processes revealed to contain potential members of the SOS cascade among their associated gene accessions. GO terms: GO:0009628 (response to abiotic stimulus), GO:0023060 (signal transmission), GO:0006812 (cation transport), and GO:0007165 (signal transduction).

Here we report a set of 13 and 7 chickpea UniTags annotated to at least 9 and 5 CIPKs and CBLs, respectively. These UniTags revealed diverse expression levels in a range between 24-fold down- and 7-fold up-regulation in salt-stressed roots, and 4-fold down- and 6-fold up-regulation in nodules, respectively. To our knowledge, no potential members of this pathway are yet known in both chickpea organs. Additionally, at least 90 UniTags distributed in 61 similar tag families (representing 47 UniProt accessions) were annotated to proton pump ATPases, proteins which can be controlled by the SOS network [67]. Concerning SOS4 and SOS5, only a single UniTag annotated to a pyridoxal kinase was detected (Q4JR83_SOYBN, STCa-4652), whereas 12 UniTags distributed in five SAAT families where annotated to AGPs. A summary of the UniTags representing the distinct CBLs, CIPKs, and AGPs is deposited in Table 5. Complete sequences of the respective homologous chickpea ESTs are deposited in Additional file 1. These transcripts may serve as starting point for validation of their function in chickpea.

**Table 5:** Summary of chickpea CBL- CIPK- and AGP-annotated UniTags in chickpea roots and nodules

	UniTag ID	Sequence	Uniprot ID	Gene class	**R**_**(ln) **_**Roots**	**R**_**(ln) **_**Nodules**
**CBL proteins**	STCa-10400	CATGCTTGTTATAGTTAGCCTTTCTC	Q5EE13_9FABA	CBL	**-0,21**	**-0,16**
	STCa-8869	CATGCCTTACTTTGGGTGTGACGATT	Q7FZ95_EUCGR	CBL	**0,89**	**-0,16**
	STCa-12426	CATGGATGTGGAAAATGAAATCCTTG	CNBLA_ARATH	CBL 10	**-0,90**	**0,53**
	STCa-7792	CATGCATATGTACTGAACCCAGTTAA	A4ZKI5_POPTR	CBL 4-1	**-2,00**	**-0,16**

**CIPK proteins**	STCa-4035	CATGAGTTTGAGATTTGTACTGTTGT	Q8LK24_SOYBN	CIPK	**0,20**	**-0,16**
	STCa-1179	CATGAAGAATCCTTGTTGATGATTCA	Q8LK24_SOYBN	CIPK	**1,99**	**-0,38**
	STCa-21948	CATGTGTTACTTGGGAGTGTCTGGTT	A0MNJ9_POPTR	CIPK 12	**1,58**	**1,63**
	STCa-19413	CATGTCAAACTACAGCAGCTGAAGCT	A0MNK6_POPTR	CIPK 19	**-1,60**	**-0,85**
	STCa-19111	CATGTATGTATGGATATATATATACT	A0MNK8_POPTR	CIPK 22	**0,89**	**0,53**
	STCa-5485	CATGATGTTATTATTTTTGATTTGAT	A0MNL1_POPTR	CIPK 25	**-0,21**	**0,53**
	STCa-21387	CATGTGGTGTTATTCTCTTTGTTCTT	A0MNL1_POPTR	CIPK 25	**0,48**	**-1,55**
	STCa-12635	CATGGCAAGGAGAACAACCACAGCAA	A0MNL4_POPTR	CIPK 6	**-2,80**	**-0,72**
	STCa-132	CATGAAAAGAATAGTGGGTAGTGTTT	CIPK7_ARATH	CIPK 7	**-0,21**	**-0,85**
	STCa-6698	CATGCAATGTAATATAAGACCCTAAT	CIPK9_ARATH	CIPK 9	**-1,19**	**1,92**

**AGP proteins**	STCa-17547	CATGTAATGTAATATTGTTGAATGAA	Q8L5F8_CICAR	AGP	**--**	**0,53**
	STCa-18898	CATGTATCATTTATTTTCTTTTTTTG	Q9XIV1_CUCSA	AGP	**1,40**	**0,53**
	STCa-17548	CATGTAATGTAATATTGTTGTGAAAA	Q8L5F8_CICAR	AGP	**0,89**	**--**
	STCa-17550	CATGTAATGTAATATTGTTGTGATTT	Q8L5F8_CICAR	AGP	**-0,12**	**0,28**
	STCa-23251	CATGTTGATGTTGGAGAGAGGGCTTT	AGP20_ARATH	AGP	**0,53**	**0,22**
	STCa-17544	CATGTAATGTAATATCGTTGTGATTT	Q8L5F8_CICAR	AGP	**0,48**	**--**
	STCa-17549	CATGTAATGTAATATTGTTGTGATTA	Q8L5F8_CICAR	AGP	**0,48**	**--**
	STCa-19513	CATGTCACAACTCATAAAAAAGTACA	Q8L5F8_CICAR	AGP	**--**	**-0,16**
	STCa-21051	CATGTGGAGTAGTTTGATTTGTGCAG	Q8L5F8_CICAR	AGP	**-0,21**	**--**
	STCa-635	CATGAAATGTAATATTGTTGTGATTT	Q8L5F8_CICAR	AGP	**--**	**-0,85**
	STCa-23101	CATGTTGAAGTGAAATATGAAATGAA	A7J385_GOSHI	AGP	**0,48**	**-0,85**
	STCa-8606	CATGCCCTCACCTCTCCTTTCATCAT	AGP25_ARATH	AGP	**-1,60**	**-1,55**

#### ROS management in salt stressed chickpea roots and nodules

The production and management of reactive oxygen species (ROS) are major early events of the stress response in plants, and consequently these features are enriched among over-represented GO terms in chickpea. ROS-scavenging is well-known from several stress contexts in plants, and therefore we will not repeat informations here [76,77]. However, filtering of the present dataset for transcript variants encoding proteins involved in ROS-related processes should identify candidate ESTs for further characterization.

As a response of plants to ROS overproduction arising from stress-induced metabolic imbalance, dismutation of superoxide (O_2_^-^) radicals by SOD occurs very quickly [78]. A total of 17 UniTags annotated to 7 SOD UniProt entries were detected in the INRAT-93 root and nodule dataset. From them, two SAAT families each with four UniTags were detected, whereas the remaining UniTags showed 1, or no associated SAAT. Belonging to one of the four-SAAT families, STCa-7896 was the most differentially expressed SOD UniTag under salt stress (40-fold up regulation in roots).

**Figure 10:**
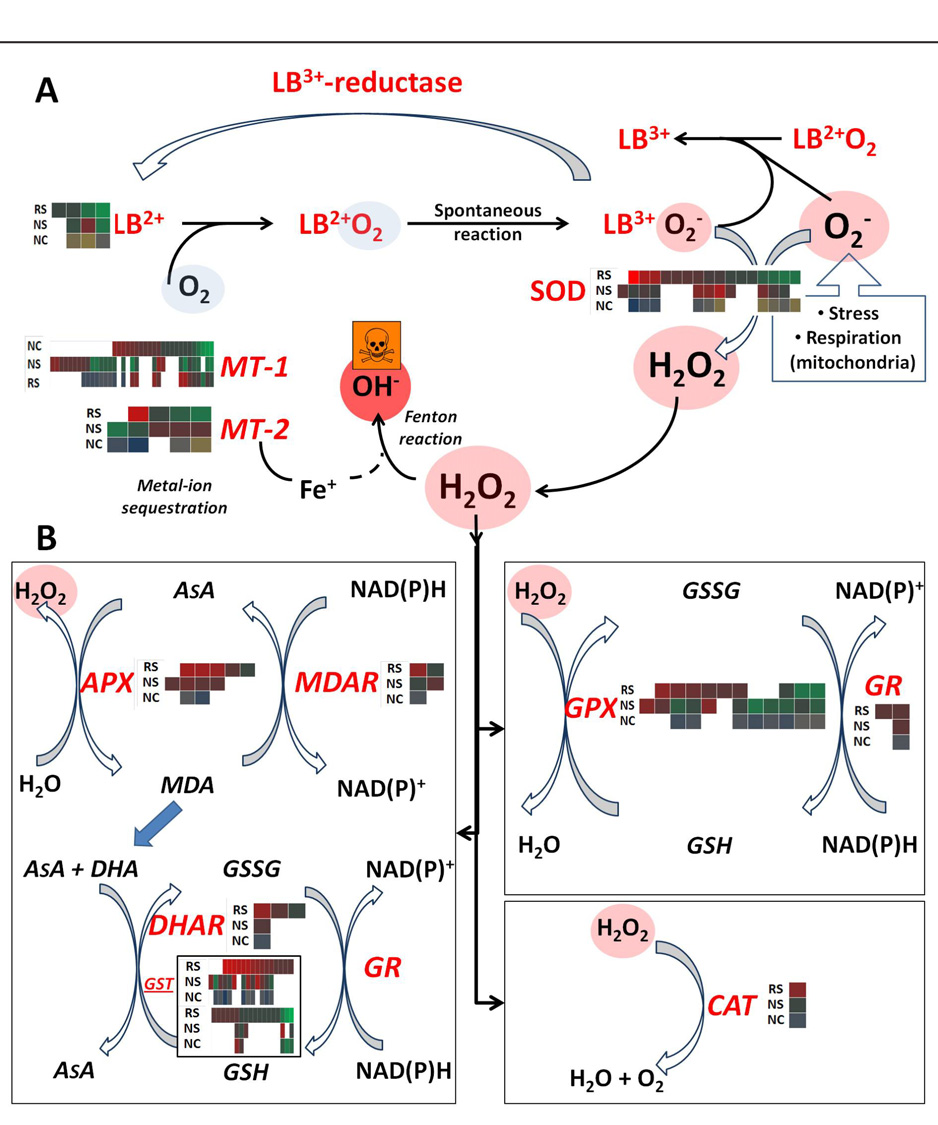
**Expression profiles of genes encoding proteins producing or detoxifying reactive oxygen species (ROS) in chickpea roots and nodules**. A) In the very intricate ROS pathway in legume nodules, superoxide radicals (O_2_^-^) are generated by elevated mitochondrial respiration rates. In turn, leghemoglobin (LB^2+^), the enzyme keeping the nodules free of molecular oxygen (O_2_), can spontaneously be converted to ferric LB (LB^3+^), generating new O_2_^-^. These radicals can induce further conversions of LB^2+ ^to LB^3+^. The generated superoxide radical can be directly dismutated by SOD to H_2_O_2_, which is immediately decomposed, as depicted in (B). On the other hand, H_2_O_2 _can generate hydroxyl radicals (OH^-^) in the presence of abundant free Fe^+ ^ions, which are sequestered by metallothionein-like proteins. B) Hydrogen peroxide can be scavenged via the glutathione/ascorbate cycles or the action of catalases (CAT) **NC: **UniTag expression profiles indicating prevalence in nodules (with various intensities of blue):Organ-specific expression **NS and RS**: UniTag expression profiles of roots and nodules, and up- and down-regulation under salt stress (with various intensities of red and green, respectively): Stress-specific expression

After dismutation of superoxide to hydrogen peroxide (H_2_O_2_), catalases (CATs), ascorbate peroxidases (APXs), dehydroascorbate reductases (DHARs), glutathione peroxidases (GPXs), glutathione reductases (GRs), and glutathione-S-transferases (GSTs) finish the H_2_O_2 _scavenging process via the ascorbate and glutathione cycles [8,9,27]. In salt stressed chickpea roots and nodules, diverse expression profiles were revealed by UniTags annotated to these enzymes. Several of these transcripts are very active in nodules even before the onset of the stress, probably due to the high metabolic activity of these chickpea organs [79]. A total of 59 UniTags annotated to proteins belonging to the ascorbate and glutathione cycles were detected in the present dataset. An overview of the plant genes involved in basic ROS-scavenging mechanisms along with the UniTag transcript levels in salt-stressed roots and nodules is depicted in Figure 10. Fold-regulation, annotation to anonymous chickpea ESTs, sequences, and information on copy numbers per SuperSAGE library for the above detailed pathways are accessible through the filtering options on Additional file 1.

#### Up-regulation of stress-related transcripts in non-treated chickpea nodules

One of the most interesting features observed on the present chickpea SuperSAGE profiles relies on the fact that several UniTags found to be prevalent in untreated nodules, become highly induced in roots after salt stress. Here, 515 and 37 UniTags resulted nodule-prevalent and salt up-regulated in roots with 3- and 8-Fold differential expression thresholds, respectively. Results that were corroborated by the GSR analysis, where at least 33 GO biological processes prevalent in untreated nodules are also over-represented in stressed roots (results previously summarized in Table 4).

To spot an example, the high representation of genes related to ROS-scavenging in non-stressed chickpea nodules, like SODs, can be explained by the metabolic demands from the nitrogen fixing machinery, whereas in salt stressed roots the high SODs induction could be triggered by a general ionic disequilibrium [28,80]. Whether this type of early induction presents any help for the plant to overcome any oncoming stress is an interesting question, not only for ROS-scavengers, but for several other categories showing similar tendencies in chickpea roots and nodules.

Previous works on poplar plants colonized by Ectomycorrhizas (EM) have already suggested that the symbiotic partnership acts as stress pre-priming event, conferring better salt stress performance to EM-colonized plants. Through a combined transcriptome and metabolome approach, Luo and co workers identified a series of common up regulated genes between EM-colonized and salt stressed poplar roots [81]. In a parallel work on drought-stressed poplar, Beniwal and co-workers found similar results [82]. On both cases, the better stress performance of EM-colonized plants involved genes acting on signal- and stress related-pathways as well as genes involved in growth and tissue structural modifications.

Despite large differences in the type of symbiotic association, plant families, and experimental conditions, a set of 6 genes sharing the same tendencies in chickpea and poplar was filtered out. Main features are deposited in Table 6. This set of genes involves ROS-, general metabolism-, nitrogen compounds transport-, and cell structure-related biological processes. The represented transcripts can be taken as candidates for further characterisation. The present work delivers already information about their expression profiles, transcript isoforms, and homologous high quality ESTs sequences.

**Table 6:** Nodule prevalent genes that were stress up-regulated chickpea roots and were equally detected in poplar roots after stress induction or EM-colonization

				Fold change			
**ID**	**Uniprot ID**	**EST ID**	**Prot Name**	**Sress roots**	**Ctr Nodules**	**Biological Process**	**N. of SAATS**

STCa-10397	G3PC_PEA	GR406687.1	G3PDH	5,68	7,63	Carbohydrate metabolism	3
STCa-1533	Q7XHJ2_QUERO	FE672553.1	Expansin-like protein	14,61	9,54	Cell. structure/Reproduction	0
STCa-22470	Q948X4_MEDSA	Contig19047	Glutathione S-transferase	12,17	19,09	ROS scavenging	0
STCa-15256	Q9FQE1_SOYBN	Contig43760	Glutathione S-transferase	8,12	7,63	ROS scavenging	4
STCa-24366	Q8L5Q7_CICAR	Contig43760	Quinone oxidoreductase	8,12	11,45	Transcription/Oxidation-reduct.	3
STCa-16259	Q9FSH3_LOTJA	Contig9994	Ammonium transporter	7,30	5,73	Transport	0

### Information transfer from SuperSAGE to other platforms

Previous reports already capitalized on the transfer of sequence information from SuperSAGE to microarrays platforms [33]. In the present study, 26 bp oligos with UniTag sequences were directly spotted onto Agilent^®^-16K arrays. In general, the signal background levels were relatively high, leading to loss of information. Nevertheless, after comparing the results from the 16K Agilent arrays with SuperSAGE profiles, a high proportion of transcripts (79%) showed shared regulation tendencies (Additional file 1). However, the signal intensities on the array did not correlate completely with the SuperSAGE expression ratios. Therefore, microarray and SuperSAGE results were confirming each other (up- or down-regulation), but were not congruent in the degree of differential expression.

One of the major drawbacks for the transfer of information from SAGE-based techniques to cDNA chips is the loss of resolution. Whereas very similar transcripts could be differentiated *in silico *by SuperSAGE, this degree of differentiation can present problems for hybridization-based techniques [83]. At present, diverse technologies have already been applied for the detection of SNPs on microarrays. However, these approaches are more directed towards genotyping than to expression profiling [84-86]. Additionally, low-abundant transcripts represent another big obstacle in the use of SuperSAGE-based microarrays. As reported for human neuronal tissue with a transcriptome full of low-abundant mRNAs, the power of microarrays is limited when "rare" messages are evaluated [87]. In the present study, most of the spotted 26 bp tags were selected on the basis of their up- or down-regulation, implying that their copy numbers are relatively high. Therefore, the microarray-based profiles do not monitor tags of low abundance. Despite the relatively good tendency congruence in both techniques for the analyzed transcripts, these drawbacks emphasize, that better strategies are still needed to improve the transference of information from deepSuperSAGE expression profiling to hybridization-based platforms.

Parallel to microarray spotting, the sequence information from the 26 bp tags was used as starting point to generate qPCR and *in situ *PCR amplifiable fragments via 3'- and 5'-RACE (data not shown). In few cases, RACE amplifications using the UniTag as starting sequence produced more than one product, which in many of the cases reflect the same homology with EST accessions shown by the original UniTags. RACE amplification of alternative, but similar ESTs is a quite common phenomenon, that could be responsible for the appearance of SNP-associated alternative tags (SAATs) in large SAGE databases [88]. Despite these technical difficulties, we transferred deepSuperSAGE-derived information to qPCR and *in situ *PCR with satisfactory results. However, hundreds or thousands of assays would be needed for a real estimation of the transfer efficiency.

## Conclusions

In the present study, deepSuperSAGE allowed to profile the transcription of genes coding for proteins involved in ROS scavenging and control of high Na^+ ^levels, among many other relevant biological processes, in various situations (roots and nodules under normal and salt stress conditions). The major insights into the prime steps of salt stress response observed in chickpea are: 1) part of the chickpea transcriptome is highly expressed in nodules already under control conditions, and a portion of it becomes highly up regulated in roots only after stress induction. 2) ROS-scavengers are highly represented among transcripts displaying this regulation tendency. This rapid activation of genes in response to salt stress was yet unknown in nodulating legumes. However, the stress-priming effect induced by symbiotic nitrogen-fixing organisms is already known from other systems, as reported for ectomycorrhizal colonization in poplar plants under saline stress [81].

For the first time at all, the magnitude of the organ- and stress-specific transcriptomes was assessed in chickpea roots and nodules. For more than 21,000 unique transcripts recovered from both organs, a clear organ-prevalence could be detected, and a stress-response level starting from 3.0-fold and going up to 20-fold differential expression was revealed.

The present report witnesses the potential of the high-throughput deepSuperSAGE technology coupled to one of the next-generation sequencing platforms (here: Roche 454 Life/APG GS FLX Titanium) for a genome-wide quantitative gene expression profiling of plants or plant organs under stress.

## Methods

### Plant treatments

Plant treatments and hydroaeroponics conditions were set according to the chickpea work of L'Taief and co-authors in which a salt concentration of 25 Mm NaCl was chosen to guarantee the functionality of the root nodules [89]. Briefly summarized, surface-sterilized seeds of chickpea cultivars INRAT-93 and Amdoun (sensitive control variety), respectively, were germinated on 0.9% agar for 5 days in dark chamber at room temperature. Seedlings with a minimum root length of 5 cm were inoculated with *Mesorhizobium ciceri *(strain UPMCa7) by dipping each seedling into growing media for 10 seconds, and packages of 15 individuals were transferred to twelve 40L hydroaeroponics buckets (6 × Amdoum, 6 × INRAT-93). Both varieties were kept in separate buckets to obtain a higher homogeneity of plant growth at intra-bucket levels. Buckets were placed alternating I-93 and Amdoum positions in a greenhouse portion showing homogeneity in light and temperature. Inter-Bucket effects were previously tested [89].

Seedlings from both varieties were further grown for 20 days in a temperature-controlled glasshouse with a day/night temperature regime of 28/20°C and a 16 h photoperiod with additional light of 400 μmol PAR m-2s-1. Micro- and macro-nutrient concentrations in the growth medium were adjusted to 0.7 mM K_2_SO_4_, 1 mM MgSO_4_^.^7H_2_O, 1.65 mM CaCl_2_, 22.5 mM H_2_PO_4 _(macronutrients), and 6.6 mM Mn^2+^, 4 mM Bo^3+^, 1.5 mM Cu^2+^, 1.5 mM Zn^2+^(micronutrients) and additionally 2.0 g L^-1 ^CaCO_3 _as pH regulator. After one round of compression and filtering, a constant air flow of 400 mL (compressed air)/liter of solution/minute was applied to each bucket through ''spaghetti" tubes system.

Three weeks-old chickpea plants were transferred to 6 new buckets with freshly prepared medium (see above), containing 25 mM NaCl (3 × I-93, 3 × Amdoum). In parallel, control plants were placed into buckets with new nutrition medium without NaCl following the same schema (3 × I-93, 3 × Amdoum). Control and 25 mM NaCl-treated roots and nodules from three randomly chosen plants extracted from three different buckets were harvested separately, and each frozen in liquid nitrogen 1 and 2 hours, respectively, after onset of the stress. Not harvested plants served to monitor changes in fresh and dry weight of INRAT-93 plants (roots, shoots, and nodules) 4 days and 5 weeks, respectively, after stress induction.

### SuperSAGE libraries

Total RNA was isolated from control and stressed roots as described by [75], except that the RNA was precipitated in 3M LiCl at 4°C overnight. The poly(A)^+^-RNA from about 1 mg total RNA was purified with the Oligotex mRNA Mini Kit (QIAGEN, Hilden, Germany) according to the manufacturer's batch protocol. Subsequent steps for construction of SuperSAGE libraries were detailed in [76]. Amplified ditags were directly sequenced on a Roche 454 Life/APG GS FLX Titanium platform.

### Data analysis

Tags of 26 bp were extracted from the sequences with the GXP-Tag sorter software (GenXPro GmbH, Frankfurt am Main, Germany, http://www.genxpro.de). Library comparisons and primary statistical treatments used DiscoverySpace 4.01 software (http://www.bcgsc.ca/ discoveryspace). Scatter plots of the expression ratios (R_[ln]_) and and respective P-values were calculated automatically by the DiscoverySpace package following the algorithm of significance for digital expression profiles from Audic and Claverie [11]. Over-representation P values for Gene Ontology (GO) categories (biological processes) observed in the different stress situations were calculated and correlated with the UniTag expression ratios (R(ln)) by applying the Gene Score Re-sampling (GSR) analysis of the ErmineJ 2.0 software package (University of British Columbia, 2006, http://www.bioinformatics.ubc.ca/ermineJ), as recommended by the software developers [90]. Additionally, cross-feedback of GSR results and stress-related pathways were carried out by constructing artificial categories containing all pathway members for the use of the ErmineJ package, as reported in [26].

### Homology searches

Tag sequences were BLASTed [77] against a total of 44,000 chickpea anonymous ESTs using the stand-alone BLAST routine. Low complexity UniTags were filtered out, and high homologous (E<1E-5) UniTag-EST hits were retained. Subsequently, all anonymous chickpea ESTs were re-annotated to different public databases discriminating the hits in a hierarchical, taxonomical manner using the BLASTN algorithm (http://www.ncbi.nlm.nih.gov/BLAST/). First, all ESTs were BLASTed against the non-redundant DNA databases, limiting the output hits with the highest priority level to *Cicer arietinum *and members of the Fabaceae, by using the routine BLASTc13 (NCBI, http://www.ncbi.org). Subsequently, individual local BLAST searches were carried out in Fabaceae sequences, followed by *Arabidopsis*, rice and maize homology searches in the TIGR gene indices (http://compbio.dfci.harvard.edu/tgi/plant.html). After each BLAST round, anonymous DNA sequences (e.g. chromosomes, shotgun clones, and ESTs not linked to any characterized protein) were filtered out. Additionally, ESTs assigned to TIGR TCs indicating weak similarity to characterized genes were not selected. The expected number of random matches (e-value) was kept under 1E-50 for individual TIGR databases larger databases (e.g. NCBI nr restricted to Fabaceae hits). Low complexity regions were rejected, whereas gap costs were set to 5-2 (NCBI BLAST standard setting).

### Rapid amplification of cDNA ends (3'-RACE)

To test the versatility of the 26 bp tag-derived oligonucleotides for direct use as 3'-RACE PCR primers, cDNA amplifications were carried out with an initial denaturation step of 94°C for 2 min, followed by 30 cycles each of 94°C for 40 sec, 55°C for 1 min, and 72°C for 1 min, with a final extension step at 72°C for 4 min. Reactions contained 15-20 ng cDNA template, 10 pmol 26 bp tag-based primer, 10 pmol oligodT (t)14-NV primer, 200 μM dNTPs, 0.4 U *Taq *DNA polymerase (Genecraft, Germany) in a buffer containing 1.5 mM MgCl_2 _supplied by the provider. After amplification, products were separated in 1.5% preparative agarose gels. Bands corresponding to unequivocal amplicons were excised, and DNA extracted with Qiaquick cleanup columns (QIAGEN, Hilden, Germany). Cloning of PCR products as well as colony PCR screening followed standard blue-white screening procedures [91]. Positive clones were sequenced via ABIprism multi-colour fluorescence-based DNA analysis system (APPLIED BIOSYSTEMS, Foster City CA, USA).

### Confirmation of SuperSAGE results by qRT-PCR

In the course of downstream applications of SuperSAGE/ST-DGE the mRNA levels of selected genes of salt stressed roots and nodules from four important cultivars of Cicer arietinum: INRAT93-1 (Beja), Amdoun-1, ICC4958 and ICC6098 were measured with qPCR assays. Total RNA was extracted following the protocol of Promega (SV Total RNA Isolation System Kit - manual TM048, http://www.promega.com/tbs/tm048/tm048.html). We elongated the DNaseI digest from original 15 minutes to 30 minutes on the column. Total RNA concentration was estimated in dilution series with LabelGuard NanoPhotometer™, IMPLEN Germany (http://www.implen.de). For the OneStep qPCR assays we applied 10-20 ng total RNA as template.

Assays for seven transcripts from the oxylipin pathway including assays for two lipoxgenase isoforms, one assay for a narbonin-like protein, and 6 assays of constitutive expressed tags (including a beta-tubulin assay - serving as invariably expressed (housekeeping) control) were developed based on SuperSAGE/ST-DGE. All assays were purchased from GenXPro GmbH, Frankfurt (see list in additional data xy). Three replicates were performed per assay, averaged and compared to the expression level of the housekeeping gene. Fold-changes in expression levels were calculated using the ∆∆-Ct method

### qRT-PCR expression profiling

Quantitative real time PCR assays (qPCR assays) used in this study were designed and provided by GenXPro GmbH, Frankfurt, Germany. The qPCR assays were run as OneStep-qPCR with dual labelled probes (FAM-BHQ) and ROX as passive reference. For each reaction of 15 μl 10-20 ng of total RNA was used as template. The OneStep-qPCR Mastermix (Clontech-Takara QTAQ-Mastermix) contains hot start Taq DNA polymerase, optimized reaction buffer, 5 mM MgCl2 (final concentration), 2.5 - 3.5 mM nucleotides (including 200 μM dUTP) and reverse transcriptase combined with an RNAse inhibitor [40 u/μl].

Specific primer were applied with a final concentration of 0.2 - 1.0 μM. The dual labelled probes had a final concentration of 0.016 - 0.08 μM. The PCR regime was done as following: i) Reverse transcription: 48°C for 20 min, ii) Activation of the hot start *Taq *DNA polymerase at 95°C for 10 min, and iii) 45 cycles with denaturing at 95°C for 15 sec, with an annealing/extension step at 60°C for one minute.

The amplification of the target genes at each cycle was monitored by qPCR probe-released fluorescence (FAM dye). The Ct, defined as the PCR cycle at which a statistically significant increase of reporter fluorescence is first detected (10× above background), was used as a measure for the starting copy numbers of the target gene. Relative quantification of the amplified targets follows the comparative ΔΔCT method. The amount of target, normalized to an endogenous reference and relative to a calibrator, is given by 2^-ΔΔCT ^[92].

### Confirmation of expression profiles by microarray hybridization

SuperSAGE expression profiles were confirmed by direct spotting of 26 bp tags onto a 16K Agilent microarray (AGILENT TECHNOLOGIES, Santa Clara CA, USA) and hybridization against fluorophore-labeled cDNAs. Three thousand UniTags with different expression levels under salt, drought and cold stresses were selected [26]. Additionally, for each of the 3,000 selected tags, oligonucleotides with mismatches were spotted onto the microarray in three sets as follows: i) mismatch at position 7; ii) mismatches at positions 7 and 13, respectively, and iii) mismatches at positions 7, 13, and 20, respectively. Background was corrected with the Feature Extraction Software™ (Agilent Technologies), subtracting the mismatch intensities for each spotted tag. Microarray design, spotting and hybridizations were carried out by ARRAY-ON GmbH, Gatersleben, Germany, according to the Agilent™ protocols (AGILENT TECHNOLOGIES, Santa Clara CA, USA).

### Sample preparation and fixation for *in situ *RT-PCR

At full flowering stage, 10 plants were selected, of which 5 were exposed to salinity (25 mM NaCl) for 2 h. Root nodules of 5 mm length were immediately harvested from the non-stressed and the salt-treated plants separately and thoroughly washed with DEPC (diethyl pyrocarbonate) treated water, then fixed in freshly prepared PFA [2% (v/v) paraformaldehyde, 45% (v/v) ethanol and 5% (v/v) acetic acid] and stored overnight at 4°C. Fixed nodules were extensively washed with four changes of DEPC-treated water over 30 min (2 × 5 min and 2 × 10 min) with agitation to remove PFA. Thereafter, the nodules were included in low melting 9% (m/v) agarose dissolved in filtered phosphate-buffered saline (PBS; 5 mM Na_2_HPO4, 300 mM NaCl, pH 7.5). The nodules in agarose blocs were cut into 50 μm thick slices using a microtome. The resulting sections were collected into small tubes containing 0.5 ml of DEPC-treated water and freed from residual agarose by three washes with DEPC-treated water heated to 60°C.

For reverse transcription, the fixed sections were transferred to PCR tubes and incubated in 40 μl RT mix [RT 1X Reaction Buffer (50 mM Tris-HCl, pH 8.3, 75 mM KCl, 3 mM MgCl_2_, 10 mM DTT) (Promega, Madison, WI, USA); 0.31 mM dNTP and 0.75 μM gene-specific reverse primer. Sequences deposited in Table 7. The samples were then heated to 65°C for 5 min, placed on ice for 2 min, then Moloney murine leukemia virus (M-MLV) reverse transcriptase H(-) (Promega) added to each sample to a final concentration of 5 U. μl^-1^, and samples were incubated at 42°C for 1 h.

**Table 7:** Ascorbate peroxidase (APX) gene-specific primer sequences for in situ RT-PCR

Gene	Forward primer	Reverse primer
**APX1**	5'-ATCCTCTCATTTTTGACAACTC-3'	5'-ACTTTTGAGTGACCCTGTATTC-3'
**APX2**	5'-ATCCTCTCATTTTTGACAACTC-3'	5'-TTTTCTTTCTTGTTGATCCTCT-3'

After reverse transcription, the RT mix was removed, and the samples were washed three times each with 100 μl DEPC-treated water. After removing the last wash, 40 μl of of PCR mix [1× PCR buffer, Invitrogen, Carlsbad, CA, USA), 1.5 mM MgCl_2_, 0.2 mM of each dNTP, 0.25 μM each of the gene-specific primer pair, 0.25 nM digoxigenin-11-2'-deoxyuridine 5'-triphosphate (Dig-11-dUTP; Roche Diagnostics, Mannheim, Germany) and 1 U *Taq *DNA Polymerase (Invitrogen)] were added. Thermocycling was performed at 95° for 3 min and 30 cycles (95°C for 30 s; 55°C for 30 s; 72°C for 45 s, 72°C for 2 min) for all the genes. Negative controls (no-RT) were prepared omitting the reverse transcription step. In this case, the samples were put in 40 μl of DEPC-treated water during the RT step, and subsequently processed as the other samples.

For the detection of the amplified cDNA, the PCR mix was removed after amplification, and the samples were washed three times each for 10 min in 200 μl PBS under gentle agitation, and then incubated in 100 μl blocking solution (2% BSA in PBS) with 0.3% Triton for 30 min under gentle agitation in darkness at 37°C.

Then the blocking solution was removed and replaced by 100 μl of alkaline phosphatase-conjugated anti-dioxygenin-Fab fragment (Roche Diagnostics) diluted 1:1000 in 2% BSA. The samples were incubated at room temperature for 1 h 30 min and then washed three times for 10 min in PBS to remove excess antibody. Detection of alkaline phosphatase was carried out using the ELF-97 (enzyme-labeled fluorescent) endogenous phosphatase detection kit (Molecular Probes, Leiden, The Netherlands). The ELF substrate was diluted 1:40 in the alkaline detection buffer (Molecular Probes, Leiden, The Netherlands), vigorously shaken, and then filtered through a 0.22-μm filter (Millex^®^-GV, Millipore, Bedford, USA) to remove any aggregates of the substrate formed during storage. Samples were incubated in 20 μl ELF substrate-buffer solution in the dark for 20 min, then washed 3 × 1 min with wash buffer (PBS with 25 mM EDTA and 5 mM levamisole, pH 8.0) before the samples were mounted. Observations were made with an Olympus BX61^® ^microscope equipped with an epifluorescence condenser, a Hoechst/DAPI filter set and a color view camera.

## Competing interests

The authors declare that they have no competing interests.

## Authors' contributions

CM and BR generated the SuperSAGE libraries and analyzed the obtained profiles. RH, FK and DS generated the 3-' and 5'-RACE sequences and designed the primers for qRT-PCR probes. MZ, JJD, and LA grew INRAT-93 in hydro-aeroponics for biological replications, as well as developed and conducted *in situ *PCR assays. GK and PW developed the experimental strategy, and were responsible for the preparation of the manuscript. All authors of the present work have read and approved the final manuscript.

## Supplementary Material

Additional file 1 **Main data matrix with UniTags annotations and expression ratios**. Sequences of UniTags-homologous chickpea ESTs. Microarray profiles from spotted UniTags Click here for file

Additional file 2 **GSR over-representation analysis results**. Raw data from each GSR independent analysis including lists of represented genes per GO term Click here for file

Additional file 3 **Potential new-uncharacterized transcripts (genes) showing high salt stress induction in chickpea roots and nodules**. Click here for file
